# Photodynamic disinfection and its role in controlling infectious diseases

**DOI:** 10.1007/s43630-021-00102-1

**Published:** 2021-10-27

**Authors:** Rafael T. Aroso, Fábio A. Schaberle, Luís G. Arnaut, Mariette M. Pereira

**Affiliations:** Chemistry Department, University of Coimbra, 3004-535 Coimbra, Portugal

**Keywords:** Photodynamic disinfection, Bacteria, Fungi, Biofilm, Virus, Inactivation

## Abstract

Photodynamic therapy is witnessing a revival of its origins as a response to the rise of multi-drug resistant infections and the shortage of new classes of antibiotics. Photodynamic disinfection (PDDI) of microorganisms is making progresses in preclinical models and in clinical cases, and the perception of its role in the clinical armamentarium for the management of infectious diseases is changing. We review the positioning of PDDI from the perspective of its ability to respond to clinical needs. Emphasis is placed on the pipeline of photosensitizers that proved effective to inactivate biofilms, showed efficacy in animal models of infectious diseases or reached clinical trials. Novel opportunities resulting from the COVID-19 pandemic are briefly discussed. The molecular features of promising photosensitizers are emphasized and contrasted with those of photosensitizers used in the treatment of solid tumors. The development of photosensitizers has been accompanied by the fabrication of a variety of affordable and customizable light sources. We critically discuss the combination between photosensitizer and light source properties that may leverage PDDI and expand its applications to wider markets. The success of PDDI in the management of infectious diseases will ultimately depend on the efficacy of photosensitizers, affordability of the light sources, simplicity of the procedures, and availability of fast and efficient treatments.

**Graphic abstract:**

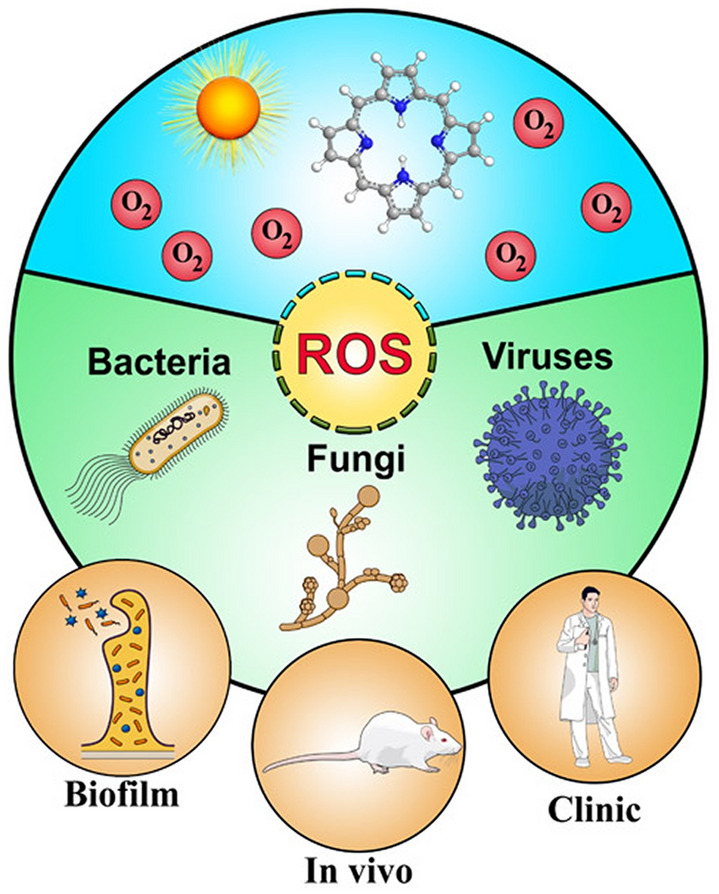

## Introduction

Infections by multi-drug resistant (MDR) microorganisms are one of the biggest challenges to healthcare systems and pharmaceutical companies, and are expected to grow considerably over the next few decades. Indeed, if no substantial developments are made in the treatment and managing of MDR infections, the number of people dying from MDR infections will jump from 700,000 in 2016 to 10 million in 2050 [1], and certainly will surpass the number of people dying from cancer, diabetes and cholera combined.

Bacterial antibiotic resistance is driven by excessive antibiotic consumption [2]. Antibiotics became widespread on the treatment of infections both in humans and in animals. The global antibiotic consumption reached 42 billion defined daily doses (DDD) for human use in 2015 and if all countries continue their antibiotic consumption rate, it will increase to 128 billion DDD in 2030 [3]. This is much aggravated by animal use. A 2014 joint European Centre for Disease Control/European Food Safety Agency/European Medicines Agency surveillance report estimated that, across 28 European Union member states, 8927 tons of antimicrobial active ingredients were used for animals, compared with 3821 tons used for medical purposes [4]. More than 70% of the antibiotics deemed medically important for human health by the FDA sold in the United States (and over 50% in most countries in the world) are used in livestock [5]. The World Health Organization (WHO) has published a list of MDR bacterial strains for which there is an urgent need for new therapeutic solutions [6]. It includes strains of Gram-negative *Acinetobacter baumannii*, *Pseudomonas aeruginosa*, *Klebsiella pneumoniae*, *Escherichia coli* and *Helicobacter pylori*, and Gram-positive *Staphylococcus aureus* and *Enterococcus faecium*.

Fungi infections are also concerning, since they result in approximately 1.5 million deaths per year, with species of *Aspergillus, Candida*, and *Cryptococcus* responsible for 90% of fungal infections in humans [7]. The prevalence of antifungal resistance is not yet at the levels observed for antibiotic resistance. However, drug-resistant fungal infections are increasingly becoming a concerning health issue, as fungal species resistant to more than one antifungal class are emerging [8]. Given that, there are only three major classes for the treatment of invasive fungal infections (polyenes, azoles and echinocandins), therapeutic options to treat multi-drug resistant fungi infections will be rapidly exhausted if antifungal resistance becomes prevalent [9]. Additionally, current antifungal therapies often give poor clinical outcomes for drug-susceptible fungal infections [10].

Viruses are the biological entity with the largest population, exhibiting high rates of mutation, and may develop resistance to antiviral therapies. This is observed when treatments are not entirely successful in inhibiting viral replication, resulting in a selective pressure that allows proliferation of resistant strains [11]. Enveloped viruses such as hepatitis C, influenza A, HIV and SARS-CoV-2 are particularly prone to mutations, and thus can more easily develop resistance to current therapies [12–14]. It is worth mentioning that exposure to antiviral drugs and metabolites in the environment may also be an important cause for antiviral resistance [15].

The demand for alternative treatments of infectious diseases originated by multidrug-resistant microorganisms is not reflected in the pipelines of pharmaceutical industries [16, 17]. In particular, the development of antibiotics effective against gram-negative bacteria, perhaps the most concerning type of MDR microorganisms, has seen no major progress in the XXI century as no new classes of antibiotics have been found. The recent clinically-approved antibiotics are derivatives of drugs for which there is widespread resistance (e.g., β-lactams and quinolones/fluoroquinolones classes) [18]. In most cases, antimicrobials have one specific biological target, given that multi-target approaches pose an increasing risk of promiscuity and can thus lead to side-effects to human cells and tissues. As a consequence, novel antimicrobials aiming at an increasingly specific target become dependent on a given mechanism, and microorganisms can more easily adapt to modify that target or block the access of the drug to the target [19]. The specificity of antibiotics is also their Achilles heel. The dilemma of antibiotic specificity/resistance or multi-targeting/toxicity is not readily solved, and fueled the revival of applications of the photodynamic effect to kill microorganisms.

The photodynamic effect was first reported in the beginning of the XX century after the observation of Oscar Raab, working in the laboratory of Hermann von Tapiener, that the illumination of microbial cultures in the presence of acridine compounds induced microbe death [20]. This discovery came when antiseptics with low toxicity to tissues, such as flavine (3,6-diamino-10-methylacridinium) were actively investigated [21] and before Fleming discovered antibiotics [22]. The large-scale use of penicillin in the Second World War promoted the uncontested use of antibiotics, and obscured the use of the photodynamic effect in the control of bacterial infections. The study of the photodynamic effect remained a minor curiosity until the 1960s, when Lipson and Schwartz gave a new impulse to the field with the demonstration that hematoporphyrin derivatives accumulate in tumors [23] and can be used as photosensitizers to destroy tumor tissue [24]. The use of the photodynamic effect in oncology met with considerable success and various photosensitizers have been approved for the treatment of solid tumors [25–28].

Today it is very well established that the photodynamic effect results from the combined actions of three elements: a photosensitizer molecule that absorbs light, a light source that emits light with a wavelength absorbed by the photosensitizer, and molecular oxygen [27]. The absorption of one photon produces one electronically-excited photosensitizer molecule initially in a singlet state but that rapidly populates a triplet state, or decays to the ground state. The lifetime of the triplet state is sufficiently long (> 1 µs) to allow for energy or electron transfer to molecular oxygen, yielding singlet oxygen or superoxide ion, respectively. Other reactive oxygen species (ROS) may be subsequently generated (e.g., hydrogen peroxide, hydroxyl radical) [29]. The triplet state of the photosensitizer may also undergo electron transfer reactions with biomolecules to generate ROS. Electron transfer reactions yield radicals and are named Type I processes, whereas energy transfer to oxygen is known as the Type II process. The use of the photodynamic effect in oncology is termed photodynamic therapy. Its use for inactivation of pathological agents such as bacteria, fungi and viruses [30–32] became known as antimicrobial PDT (aPDT), photodynamic inactivation (PDI), photodynamic antimicrobial chemotherapy (PACT) or photodynamic disinfection (PDDI). Recently, the conjugation of a photosensitizer molecule with a monoclonal antibody that targets an expressed antigen on the cancer cell surface has been referred as photoimmunotherapy (PIT) [33]. Although different designations are employed in different fields, the nature of the photodynamic process is the same.

The mechanism of cell death triggered by the photodynamic effect depends on the oxidative stress locally produced [34]. The ROS generated in PDDI (singlet oxygen ^1^O_2_, superoxide ion O_2_^⋅–^, hydrogen peroxide H_2_O_2_, hydroxyl radical OH^⋅^), have relatively short lifetimes and react with biomolecules before having time to diffuse from the illuminated area. The lifetime of singlet oxygen (*τ*_∆_) in cells and its associated diffusion length were recently established: *τ*_∆_ ≈ 3 µs [35], i.e. a diffusion length of 200 nm over a period of 5*τ*_∆_. The hydroxyl radical is extremely reactive and its lifetime in cells is 1 ns, which limits the radius of the volume where it can produce damage to 1 nm [36]. Superoxide ion and hydrogen peroxide are natural by-products of cellular metabolism. It is estimated that the aggregate rate of H_2_O_2_ formation inside aerobic *E. coli* is 10–15 µm/s [37] and that of O_2_^⋅–^ is 5 µm/s [38]. The toxicities and fast rates of formation of these ROS led cells to developed specialized scavenger enzymes and stringent antioxidants (e.g., glutathione, cysteine). Catalases and peroxidases keep the steady-state concentration of H_2_O_2_ in cells at ~ 20 nM [37]. Superoxide dismutases are sufficiently abundant in the cytoplasm to keep O_2_^⋅–^ at ~ 0.2 nM [38]. The diffusion of O_2_^⋅–^ is also limited by its poor ability to cross biological membranes. The relatively high diffusion radius of singlet oxygen and the lack of specialized endogenous scavengers to control its concentration, combine to make Type II processes particularly important in PDDI of microorganisms. Singlet oxygen reacts with proteins, nucleotides and lipids with rate constants of ~ 10^4^, ~ 10^3^ and ~ 10^2^ L/(g s). Considering the relative abundance of protein in cells, it is expected that quenching of singlet oxygen by proteins is two orders of magnitude higher than by nucleotides and lipids combined. Hence, when PDDI is performed with the photosensitizer inside the cell, proteins are likely the primary target of singlet oxygen. However, if PDDI is performed before the photosensitizers have time to permeate cell membranes, the oxidation or peroxidation of lipids may become determinant in the inactivation of microorganisms. The higher solubility of singlet oxygen in lipids than in aqueous environments, and the higher proportion by mass of lipids in the membrane, also contribute to make biological membranes attractive targets in PDDI. The diversity of ROS and their high reactivity towards different biomolecules ensures that PDDI is a multi-target approach to control infectious diseases, which reduces the efficacy of drug resistance mechanisms [39]. Moreover, PDDI is applied for a short period of time (typically the illumination lasts for just a few minutes) and it is uncommon to systematically repeat PDDI over long treatment periods. Taken together, these factors explain why the magnitude of resistance to the photodynamic effect is less than that observed for chemotherapy and antibiotics [40, 41].

Although PDDI attains multiple cellular targets, it benefits from the directionality of light to minimize off-target damage. This also contributes to make PDDI especially suitable to treat localized infections [42]. The photosensitizer can be applied locally and, after a proper drug-to-light interval (DLI), the light dose is delivered to the infected area. Examples of localized infections include periodontal diseases, burn infections, surgical wound infections and infected wounds originated by venous, pressure or diabetic ulcers [43–45]. Superficial wounds are defined as wounds that affect only the epidermis. The epidermis reaches a maximum thickness of ~ 1.5 mm on the palms of the hands and the soles of the feet. Superficial wounds, including stage I pressure ulcers and stage 0 diabetic ulcers, are particularly suited for PDDI with topical administration of photosensitizers. Partial-thickness wounds extend through the epidermis into, but not through, the dermis, and correspond to depths between 1 and 4 mm. This is the case of stage II pressure ulcers. The slow diffusion of the topically-applied photosensitizer through the epidermis and low optical penetration depth of light at wavelengths shorter than 650 nm may become limiting factors in PDDI of infected partial-thickness wounds. Full-thickness wounds extend through the epidermis and dermis into subcutaneous fat and deeper structures. They correspond to stage III pressure ulcers, venous ulcers or surgical wounds [46, 47]. These wounds are open wounds and light and photosensitizer do not have to penetrate 4 mm or more into the skin to reach the infection. Nevertheless, the clinical presentation of such large wounds may require debridement and this may still leave obstacles to homogeneous illumination and photosensitizer delivery. In addition to wound infections [48, 49], other possible superficial targets of PDDI include acne [50] (i.e., colonization of follicles by *Propionibacterium acnes*) and impetigo [51] (mostly caused by *Staphylococcus aureus*, which colonizes the nasal epithelium first and from this reservoir colonizes the skin). Superficial soft tissue infections of the ear, nose and throat/upper respiratory tract (e.g., tonsillitis, pharyngitis, scarlet fever, otitis media, sinusitis) may also be controlled by PDDI [52, 53].

Although localized infections are the most obvious therapeutic indication for PDDI, the photodynamic pathogen inactivation of single units of fresh frozen plasma met with considerable commercial success in Europe. Initially developed to increase the viral safety of plasma transfusions and more recently shown to inactivate bacteria in plasma, PPDI of plasma with methylene blue has been used to treat more than 6 million plasma units in the last 15 years [54]. Other methods of extracorporeal blood photodisinfection are emerging to treat systemic infections such as sepsis [55]. We can expect to see continued advances in such methods but, for the purposes of this work, we will focus on recent development of PDDI that can potentially translate to the treatment of localized infections.

In 2004 Hamblin and Hasan authored a very impactful review on the use of PDDI to treat infections [31]. This followed from research on PDDI using polycationic photosensitizer conjugates that remains inspiring [56]. Hamblin’s contributions to PDDI also include the disclosure of very potent photosensitizers with intense absorption in the near-infrared [57], important animal models to refine PDDI approaches [58, 59], and methods to potentiate the efficacy of PDDI [60], among very numerous other contributions [31, 42, 61–64]. It is a great pleasure to contribute to a special issue celebrating the achievements of Mike Hamblin with this review on PDDI.

Various excellent reviews on PDDI have been published [32, 65–77], including with a focus on the treatment of multi-resistant bacteria in planktonic suspensions or in biofilms [78], as well as fungi [79] and viruses [80, 81]. Many efforts have been dedicated to the synthesis of photosensitizers and to new strategies for PDDI [62, 64, 66, 67, 82, 83], including the combination with antimicrobials [32, 84, 85]. There is also an interesting literature on the use of blue light (400–450 nm) to excite endogenous photosensitizers that generate oxidative stress or to produce oxygen-independent DNA damage [86], but such approaches are intrinsically limited by the low penetration of blue light in human tissues. Our approach in this work is to focus on photosensitizers that have been applied to clinically-relevant systems (e.g., biofilms, animal models of infection) or that are employed in the clinic, identify their factors of success and relate them with properties of the systems. Success in PDDI also depends on proper choice and use of light sources. A detailed analysis of light delivery to infectious diseases and of available light sources is also presented. To understand better the specificities of photosensitizers aiming at the inactivation of microorganisms, a very brief overview of photosensitizers employed in clinical PDT of solid tumors is presented to set the stage for photosensitizers used in PDDI of microorganisms.

## PDT of solid tumors

It was emphasized above that even the ROS with the largest diffusion length (~ 200 nm for ^1^O_2_) deactivates within a very small volume. Indeed, ^1^O_2_ explores a radius ca. 2 orders of magnitude smaller than that of a typical a human tumor cell. This means that cell death triggered by PDT oxidative stress is facilitated if the photosensitizer first enters the tumor cell. Lower photosensitizer doses are required to kill tumor cells if they are exposed to light after substantial photosensitizer uptake.

**Fig. 1:**
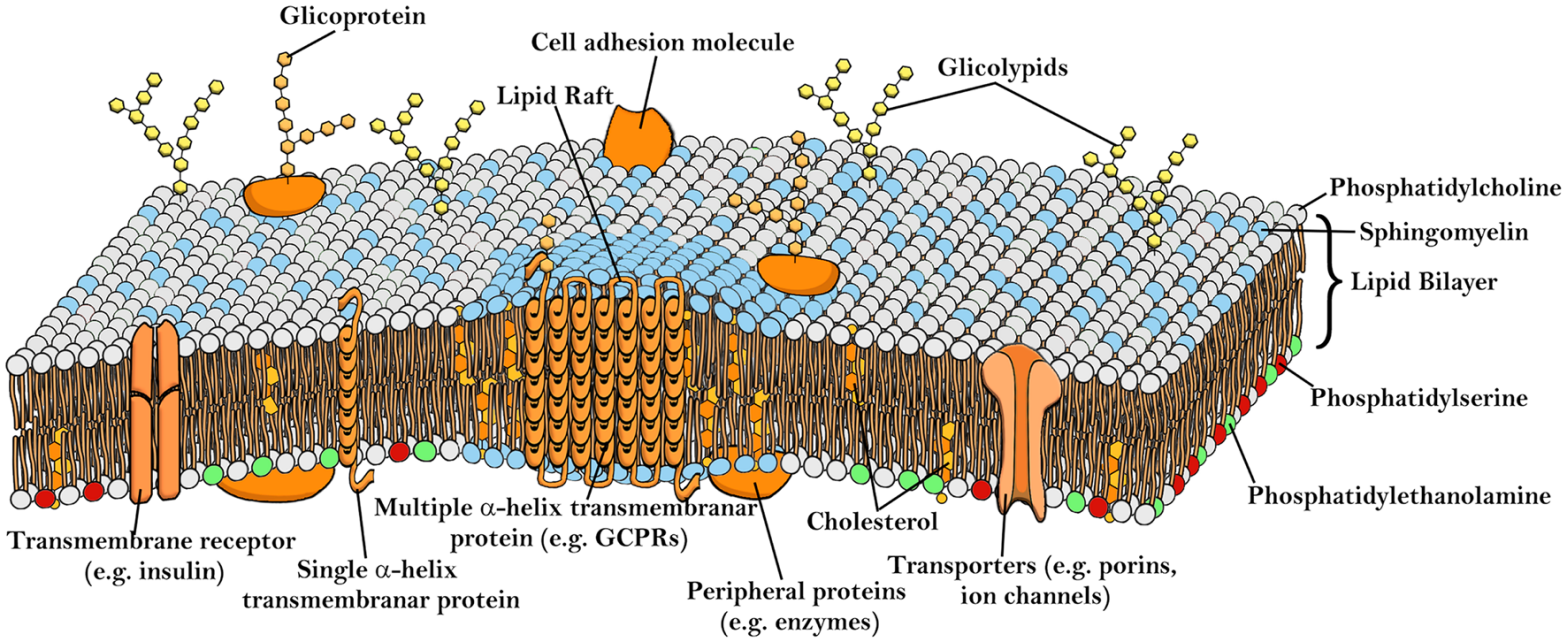
Schematic representation of the biological membrane in animal cells [89]

Human cell membranes, illustrated in Fig. 1, are particularly well studied [87] and only a brief description is needed in the context of photosensitizer cell uptake. The structural basis of human cell membranes is a complex lipid bilayer, constituted mostly by phosphatidylcholine, sphingomyelin, cholesterol, phosphatidylethanolamine, and phosphatidylserine [88]. The distribution of these constituents in the inner and outer leaflets is heterogeneous, as phosphatidylethanolamine and phosphatidylserine are more prominent in the inner leaflet, while sphingomyelin and phosphatidylcholine are located mainly in the outer leaflet. Figure 1 also depicts the lipid rafts, which are membrane microdomains more ordered and tightly packed than the rest of the bilayer, and contain high amounts of cholesterol and sphingomyelin [89]. Embedded in the lipid bilayer are peripheral and transmembrane proteins that serve multiple purposes, namely as enzymes, transporters, receptors and cell adhesion molecules. In addition, polysaccharide chains located in the extracellular environment and linked to lipids (glycolipids) and proteins (glycoproteins) forming the glycocalyx, play an important role in immune response namely in cell recognition, cell–cell interactions and protection from the environment.

The transport of substances through cell membranes may occur by simple diffusion, facilitated diffusion with the aid of a membrane protein, or active transport with an energy penalty paid by the cell. The latter two transport mechanisms are endocytic pathways. It could be desirable to avoid endocytosis, and the associated low cytosolic release of the transported substance, and have photosensitizers that passively diffuse across the membrane, driven by a concentration gradient. However, the derivation of Fick´s first law of diffusion for passive diffusion across a plasma membrane gives [90]

$$\frac{\mathrm{d}M}{\mathrm{d}t}=\frac{{P}_{M}S\Delta C}{d\sqrt{MW}}$$

where d*M*/d*t* is the amount of compound transferred across a membrane over time *t*, *P*_M_ is the membrane-water partition coefficient of the compound, *MW* is the molecular weight of the compound, ∆*C* is its difference in extra/intracellular cellular concentration, *S* is the total surface area of the membrane, *d* is the thickness of the membrane. Only low *MW* drugs may diffuse rapidly across cell membranes. This is also pictured by Lipinski’s “rule-of-five”, which describes the bioavailability of oral drugs [91]. One of the Lipinski’s rules states that drugs should have *MW* ≤ 500 Da for high bioavailability after oral administration. This bioavailability and membrane permeability are closely related because drugs traverse the gut epithelium mostly by transcellular transport.

Photosensitizers employed in PDT of cancer have been extensively reviewed [27, 28, 70, 92–95]. Our perspective here is to recall some examples of photosensitizers in clinical use, including photosensitizers in clinical trials, to emphasize some of their properties and their implications in translation to infectious diseases. All the photosensitizers in Table 1 have *MW* > 650 Da. It is not surprising that they require intravenous administration with appropriate formulations. With the exception of cetuximab saratolacan, their size and hydrophobicity favors endocytosis mediated by low density lipoproteins receptors as the main mechanism of cell uptake [27], although temoporfin shows some degree of simple diffusion through the cell membrane [96]. The main cellular compartments where these lipophilic compounds accumulate include mitochondria, endoplasmic reticulum, Golgi apparatus, nuclear and lysosomal membranes [97, 98]. Cetuximab saratolacan is an antibody–drug conjugate that targets the epidermal growth factor receptor often overexpressed on the surface of cancer cells. When this photosensitizer is excited, it releases ligands causing stress in the cellular membrane, impairing its function and leading to cell death [99]. This photosensitizer is not internalized by the cells.

**Table 1:** Chemical properties of main tetrapyrrolic macrocycles used in PDT under clinical use

#	Name/application	Structure	*MW*, g/mol	Log *P*_OW_	Charge
1	Porfirmer sodium (Photofrin) [100]		1179 (for *n* = 0)	0.15	− 4 (for *n* = 0)
2	Temoporfin (Foscan) [101] Head and neck		680	5.5 [102]	0
3	Verteporfin (Visudyne) [103] Pancreatic cancer [104]		718	1.6	− 1
4	Talaporfin sodium Lung cancer [105]		712	− 3 [106]	− 4
5	Padeliporfin (Tookad-soluble) Prostate cancer [107]		872	− 0.2	− 2
6	Redaporfin Head and neck [108]		1135	1.9	0
7	Cetuximab saratolacan (Akalux) Head and neck [33]		156,000–158,000	–	− 4 (excluding cetuximab)

Cell uptake is relatively slow (> 24 h to reach the maximum) for porfimer sodium and for redaporfin, and significantly faster (2–3 h) for temoporfin and verteporfin [96, 108, 109]. This follows the expectations based on their molecular weights. The cell uptake of talaporfin is also relatively slow (> 4 h) [110], certainly because of the combination between its moderately high molecular weight and 4 negative charges. Cell uptake and pharmacokinetics help explain why these photosensitizers are usually employed either at long drug-to-light intervals (“cellular”-PDT, DLI > 24 h for porfimer sodium and temoporfin) or at short DLI (“vascular”-PDT, DLI < 30 min for verteporfin, padeliporfin, redaporfin). Talaporfin is employed in early-stage lung cancer with DLI = 4–6 h and in brain tumors with DLI = 22–26 h. The short DLI employed in PDT with verteporfin, padeliporfin and redaporfin target the photosensitizers while in the vascular compartment, rather than inside tumor cells. PDDI would be most appealing at short DLI, but it is not possible to use vascular effects to selectively inactivate microorganisms. Photosensitizers for PDDI must be based on different molecular designs.

PDT of actinic keratosis met with considerable success when precursors of Protoporphyrin IX (PpIX) such as 5-aminolevulinic acid (5-ALA, commercialized as Levulan^®^ in the USA) or 5-aminolevulinic acid methyl ester (MAL, commercialized as Metvix^®^) were administered in topical formulations. 5-ALA has a molecular weight of only 131 g/mol and its methyl ester MAL increases the molecular weight to 145 g/mol. These small molecules diffuse rather rapidly through the skin and are efficiently internalized by cells. A randomized, double-blind, prospective study to compare Levulan^®^ and Metvix^®^ in PDT of extensive scalp actinic keratosis showed that there is no significant difference in efficacy between them [111]. Interestingly, this comparative study employed a red light (580–740 nm) for both products, but Levulan^®^ is indicated for use with BLU-U Blue Light PDT Illuminator (417 nm) whereas Metvix^®^ employs Aktilite CL (630 nm). These peak wavelengths match the Soret band and the lowest energy band of PpIX, respectively.

The use of blue light in PDT of actinic keratosis may be surprising since it is known that the penetration of light in human skin increases with the wavelength. The optical penetration depth increases from *δ* ≈ 0.3 mm at 417 nm to *δ* ≈ 1.7 mm at 630 nm [112]. This means that 2 mm beneath the surface of the human skin, light intensity at 417 nm is attenuated by a factor of 1.3 × 10^–3^ whereas at 630 nm it is only reduced by a factor of 0.3. The increase of optical penetration depth in the red/infrared can have dramatic consequences in the treatment of thick solid tumors and motivated the development of photosensitizers with intense absorptions in the phototherapeutic window, i.e., between 650 and 850 nm [27]. However, the photodynamic effect comes from the number of photons absorbed, and this depends both on the number of photons available at 417 and 630 nm and on the absorption coefficients of the photosensitizer at these wavelengths. The ratio of the absorption coefficients of PpIX at 410 vs. 624 nm in cells is ~ 56 [113], which partly compensates the poor penetration of blue light in the skin. This comparison between Levulan^®^ and Metvix^®^ teaches that when the therapeutic target is within 2 mm of skin surface, the lower tissue penetration of light with wavelengths shorter than the phototherapeutic window can be partly compensated by high absorption coefficients.

In summary, photosensitizers for PDT of cancer are designed to have intense absorptions above 650 nm and may be rather large “macromolecules” administered by intravenous injection. Their molecular size is not critical for success because they may operate via a vascular shutdown or by impairment of the cell membrane, and these mechanisms allow for solid tumor destruction without photosensitizer internalization by tumor cells. Moreover, when cellular-PDT is desired, a long DLI can be employed to allow the photosensitizers to be internalized by the cells. The clinical adoption of photodynamic disinfection for the treatment of superficial infections requires a topical application of the photosensitizer followed within a few minutes by illumination of the infected area. Wavelengths in the visible range may be effective if the absorption coefficient of the photosensitizer is high, it can diffuse rapidly into the infected tissue, and the depth of the treatment does not need to exceed 3 mm. Clearly, the development of photosensitizers for PDT or for PDDI is not driven by the same requisites.

## Bacteria and biofilms

The goal of PDDI must be to cure the local infection. When dealing with bacterial infections, it is important to distinguish between the bacteriostatic effect defined as the effect of an agent that prevents the growth of bacteria (i.e., keeps the bacteria in the stationary phase of growth), from the bactericidal effect where the agent kills the bacteria [114]. In practice, it can be considered that an agent has a bacteriostatic effect if it inhibits bacterial growth 24 h post-treatment, with less than a 99.9% decrease in the number of colony forming units (CFU). A bactericidal effect requires at least a 3 log (99.9%) CFU reduction. The potency of antibiotics is often characterized by two measures: (1) their minimum inhibitory concentration (MIC), i.e., the lowest concentration that results in inhibition of bacterial growth after 24 h incubation; (2) their minimum bactericidal concentration (MBC), i.e., by the lowest concentration that results in 3 log CFU reduction. This measure is not entirely adequate for photosensitizers because lower drug concentrations can be partly compensated by higher light doses, and the incubation times relevant for photosensitizers (less than 1 h) and for antibiotics (18–24 h) are widely different. The discussion of the light doses is postponed to Sect. 6. Nevertheless, photosensitizers with bactericidal effects that require photosensitizer concentrations higher than 50 µM are likely to be difficult to translate to clinical practice because such high concentrations will be difficult to achieve in the whole infected region and may be toxic to human cells.

### Biological barriers in bacteria and biofilms

**Fig. 2:**
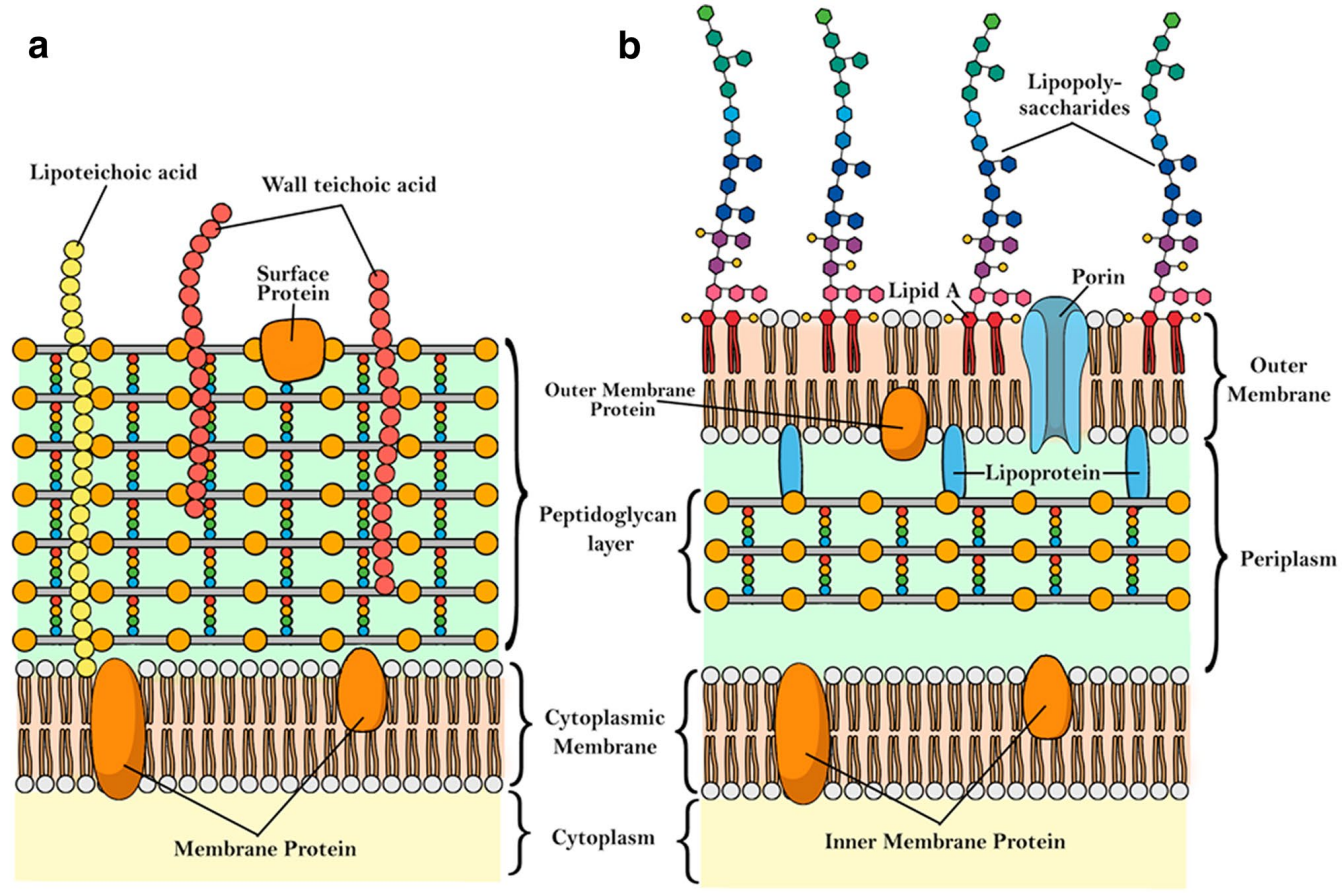
Schematic representation of the biological membranes in bacteria: **a** Gram-positive bacteria; **b** Gram-negative bacteria [115]

It is now understood that only photosensitizer molecules located in the cellular membrane or inside the cells, are able to generate ROS that can damage cell components and lead to cell death. This gives special relevance to the understanding of the biological barriers that the photosensitizers must cross before reaching their targets. There are two types of barriers that are relevant to photosensitizers targeting bacteria: the cell wall and the bacterial biofilm. Gram-positive (G+) and Gram-negative (G−) bacteria have substantially different cytoplasmic membranes, as shown in Fig. 2.

The membranes of G+ bacteria are characterized by a 15–80 nm thick layer of up to 100 peptidoglycan chains that retains crystal violet stain after it is washed from the sample in the Gram stain test. The cytoplasm is surrounded by a single lipid bilayer, composed mainly by phosphatidylglycerol (~ 70–80%) and cardiolipin (~ 20%) [116], in which some proteins are embedded. Facing the outer leaflet are multiple strands of peptidoglycan stacked one upon another and cross-linked for additional strength. Inside the peptidoglycan layer there are copolymers of glycerol phosphate or ribitol phosphate and carbohydrates, linked via phosphodiester bonds, called teichoic acids (if bound to peptidoglycan layer) or lipoteichoic acid (Fig. 2a). This layer has a high degree of porosity that allow large macromolecules to diffuse readily to the cytoplasmic membrane [69]. In addition to the multidrug resistant G+ bacteria already mentioned, other clinically-relevant G+ bacteria are: *Streptococcus pneumoniae*, *Streptococcus epidermis*, *Streptococcus mutans*, *Enterococcus faecalis* and *Propionibacterium acnes*.

The walls of G− bacteria are composed by an inner phospholipid bilayer, followed by a peptidoglycan layer, which anchors the outer membrane bilayer through lipoproteins. The phospholipid inner bilayer is composed by 80% of zwitterionic phosphatidylethanolamine, ~ 15% of anionic phosphatidylglycerol and ~ 5% of anionic cardiolipin [117]. The outer membrane possess an additional lipid bilayer (10–15 nm thick) above the peptidoglycan network, that includes lipopolysaccharides, rich in negatively charged phosphate groups, consisting of a lipid portion (lipid A) linked to polysaccharides [118] and proteins with porin function (Fig. 2b). The lipopolysaccharides, which carry a net negative charge, are non-covalently cross-bridged by divalent cations such as Ca^2+^ and Mn^2+^ [119]. This membrane structure is one of the stringent limitations for antibiotic treatment of G− bacteria since only relatively small molecules (*MW* < 600 Da) can diffuse through the porin channels [69, 117]. Large antibiotic molecules, such as colistin, are able to disrupt negatively charged membranes, but include an amphiphilic moiety to enhance the interaction with the membrane [120]. In addition to the multidrug resistance G− bacteria already mentioned, *Porphyromonas gingivalis* is also clinically relevant [121].

**Fig. 3:**
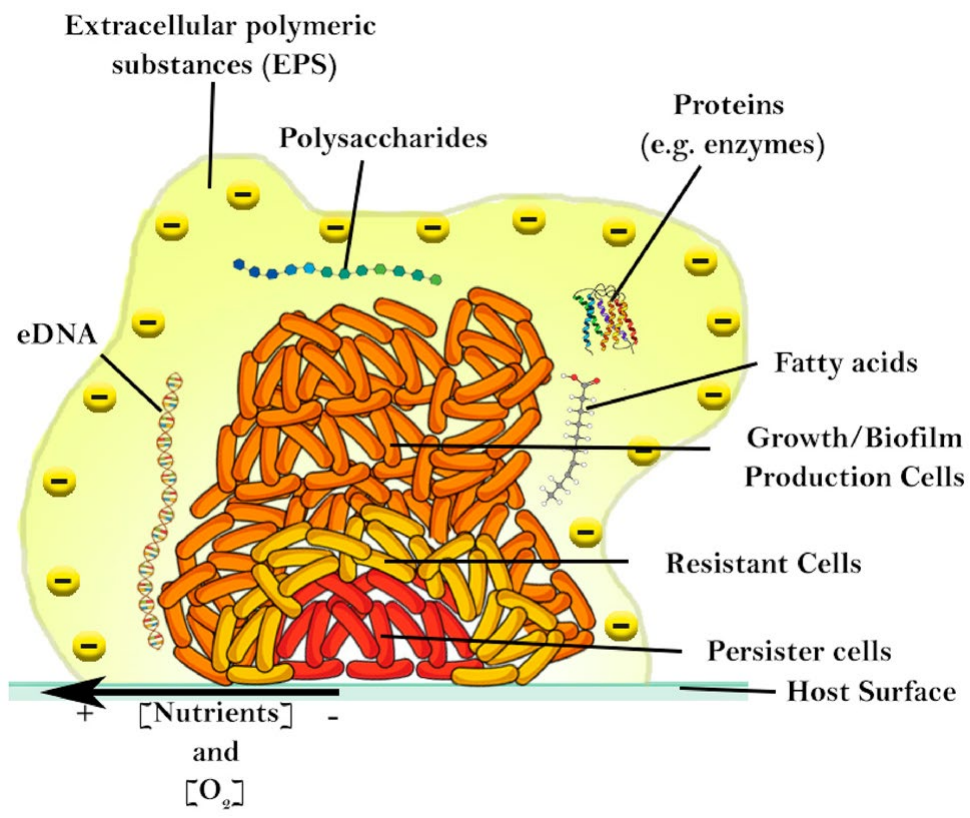
Schematic representation of bacterial biofilm [124]

Most PDDI studies are performed with bacteria in planktonic form. However, the vast majority of bacterial infections, and particularly those associated with chronic infections, are caused by bacteria in the form of biofilms [122], which are 10 to 1000 times more difficult to destroy than planktonic bacteria [62]. A biofilm is a community composed of bacteria from single or multiple species, capable of various phenotypic transformations, to perform different functions. It consists in multiple layers of cells embedded in a negatively charged matrix composed by extracellular polymeric substances (EPS) englobing extracellular DNA (eDNA), polysaccharides, proteins (e.g., enzymes) and fatty acids [123] (Fig. 3). These polymers create the first barrier of protection, as they bind to positively-charged antibiotics, preventing their diffusion to the core. The high viscosity and low permeability of bacterial biofilms create a gradient of nutrients and oxygen, with low quantities in the core. This means that the activity of antibiotics is also affected since only low quantities reach the interior of the biofilm [124]. Therefore, in the core we find persister and resistant bacteria. These bacterial core cells are in a higher dormancy state, which lowers their metabolism and, consequently, their nutrient requirements. Since most antibiotics target metabolic and cell division pathways, it is not surprising that these bacteria are inherently more resistant to antibiotics. Despite this dormant state, if the upper layers of the biofilm are destroyed, bacteria can awake and rebuilt the biofilm, leading to relapses in the treatment of bacterial infections. While some studies suggest that persister cells are susceptible to ROS inactivation [125], the lower oxygen concentration at the biofilm’s core may hinder the success of PDDI. In this regard, some strategies have been proposed to reduce biofilm hypoxia, such as hyperbaric oxygen therapy [126], the use of MnO_2_ nanosheets which catalyze O_2_ formation from H_2_O_2_ [127] or O_2_-carrying perfluorohexane-loaded liposomes [128]. In particular, this last approach has recently been successfully employed in the treatment of bacterial keratitis in rat cornea [129].

The existence of a negatively charged matrix composed by extracellular polymeric substances determines the development of new antibiotics and the design of new photosensitizers where size and charge matter [71, 82, 130]. In view of the relevance of biofilms and of the challenges they present for the development of better photosensitizers, this work will address the photodynamic disinfection of biofilms rather than of planktonic bacteria.

### Photodisinfection of bacterial biofilms

Tables 2 and 3 summarize recent studies (2015–2020) on in vitro photoinactivation of bacteria in biofilms and refer to G+ and G− bacteria, respectively. In general, more photosensitizers achieve a bactericidal effect with G+ bacteria than with G− bacteria. It is widely recognized that the difficulty to kill G− bacteria is closely related to the structure of its cell wall, which is more difficult to penetrate than that of G+ bacteria.

**Table 2:** Chemical properties and biological activity of photosensitizers used in vitro assays for inactivation of bacterial biofilms of Gram-positive bacteria

#	Structure	Biofilm strain	Results
1	IP-2-Zn (PS**1**) Charge: + 2 *MW* = 592 Da	*S. aureus* ATCC 25925 [82]	Outcome: 7 log CFU reduction [PS] = 5.2 nM Light dose: 5 J/cm^2^ (Biotable *λ* = 400–650 nm)
2	IP-4-Zn (PS**2**) Charge: + 4 *MW* = 754 Da	*S. aureus* ATCC 25925 [82]	Outcome: 6 log CFU reduction [PS] = 1 µM Light dose: 12 J/cm^2^ (Biotable *λ* = 400–650 nm)
3	Porphyrin mixture FORM (PS**3**) Charge: + 1 to + 4 *MW* = 679 to 900 Da	MRSA DSM 25693 [131]	Outcome: ~ 7 log CFU reduction [PS] = 1.0 µM (in combination with 100 mM KI) Light dose: 9 J/cm^2^ (white fluorescent lamp *λ* = 380–700 nm)
4	PS**4** (in polymeric micelles of Pluronic F-127) Charge: + 4 *MW* = : 1384 Da	*S. aureus* 209P [132]	Outcome: > 99% bacterial death [PS] = 10 µM Light dose: 128 J/cm^2^ (500 W halogen lamp with filter *λ* = 420–1000 nm)
5	TAPP (PS**5**) Charge: 0 to + 4 (pH dependent) *MW* = 1019 Da	*S. aureus* RN6390 [133]	Outcome: 4 log CFU reduction [PS] = 50 µM Light dose: 180 J/cm^2^ (Tungsten Halogen lamps 500 W)
6	TAPC (PS**6**) Charge: 0 to + 4 (pH dependent) *MW* = 1021 Da	*S. aureus* ATCC 25923 [134]	Outcome: 4 log CFU reduction [PS] = 10 µM Light dose: 108 J/cm^2^ (Novamat 130 AF slide projector 150 W lamp, using optical filters *λ* = 350–800 nm)
7	Chlorin e6 (PS**7**) Charge: − 3 *MW* = 597 Da	Multi-species biofilm: *M. catarrhalis* strain 7169, NTHi strain 86-028NP, *S. pneumoniae* EF3030 [135]	Outcome: 3–7 log CFU reduction [PS] = 10 mM Light dose: 123 J/cm^2^ (LED *λ* = 405 ± 10 nm)
8		*S. mutans* UA159 [136]	Outcome: ~ 5 log CFU reduction [PS] = 200 µM Light dose: 15 J/cm^2^ (LED *λ* = 660 nm)
9	Photodithazine (PS**8**) Charge: − 2 *MW* = 581 Da	*S. mutans* UA159 [137]	Outcome 4.6 log CFU reduction [PS] = 1 mM (in combination with 1.7 mg/mL chitosan suspension) Light dose: 39.5 J/cm^2^ (Laser *λ* = 660 nm)
10	5-ALA: protoporphyrin IX precursor (PS**9a**) Charge: 0 *MW* = 131 Da	*S. epidermis* (clinical isolate) and *S. aureus* ATCC 25923 [138]	Outcome: 5 and 6 log CFU reduction, respectively [PS] = 2 mM ALA Light dose: 210 J/cm^2^ (Tungsten Halogen lamps 500 W)
11	MgPc-octamorph (PS**10**) Charge: + 8 *MW* = 1690 Da	*S. aureus* NCTC 4163 [139]	Outcome: 3.3 /2.97 log CFU reduction [PS] = 1 mM/100 µM Light dose: 1.9 J/cm^2^ (visible light)
12	PS**11** Charge: + 4 *MW* = 1235 Da	*S. aureus* CMCC 26003 [140]	Outcome: 2 log CFU reduction [PS] = 0.5 µM Light dose: 24 J/cm^2^ (laser light)
13	Hypericin (PS**12**) Charge: 0 *MW* = 504 Da	*E. faecalis* ATCC 29212 [141]	Outcome: 0.4 log CFU reduction [PS] = 15 µM Light dose: 12 J/cm^2^ (40 yellow LEDs *λ* = 590 ± 10 nm)
14	Riboflavin (PS**13**) Charge: 0 *MW* = 376 Da	MDR *S. aureus* (clinical isolate) [142]	Outcome: 0.3 log CFU reduction [PS] = 50 µM Light dose: n.a. (LED *λ* = 450 nm; 40 W/cm^2^)
15	Curcumin (PS**14**) Charge: 0 *MW* = 368 Da	*S. aureus* ATCC 25923 and ATCC 33591 [143]	Outcome: ~ 3.4 and 2.0 log CFU reduction, respectively [PS] = 80 µM Light dose: 5.28 J/cm^2^ (LED *λ* = 455 nm)
16	Curcumin (PS**14**) (imobilized in endotracheal tubes)	*S. aureus* ATCC 25925 [144]	Outcome: 1.3 log CFU reduction [PS] = 0.5% w/w Light dose: 50 J/cm^2^ (LED *λ* = 450 nm)
17	Curcumin (PS**14**) + Phycocyanin (PS**15**) (in chitosan nanoparticles)	*S. aureus* KY770792.1 [145]	Outcome: 1.1 log CFU reduction [PS] = 100 µg/ml Light dose: 10 min with 5 W LED lamp
18	SAPYR (PS**16**) Charge: + 1 *MW* = 272 Da	*S. mutans* ATCC 25175 [146]	Outcome: 6 log CFU reduction [PS] = 50 µM Light dose: 30 J/cm^2^ (gas-discharge lamp *λ* = 380–600 nm)
19	SAPYR-PN-05 (PS**17**) Charge: + 1 *MW* = 407 Da	*S. mutans* ATCC 25175 [146]	Outcome: 6 log CFU reduction [PS] = 500 µM Light dose: 30 J/cm^2^ (gas-discharge lamp *λ* = 380–600 nm)
20	Methylene blue (PS**18**) Charge: + 1 *MW* = 284 Da	*S. aureus* ATCC 29213 [147]	Outcome: 6 log CFU reduction [PS] = 200 µM Light dose: 18 J/cm^2^ (LEDs *λ* = 625 ± 10 nm)
21	New methylene blue (PS**19**) Charge: + 1 *MW* = 312 Da	*E. faecalis* MTCC 2729 and *K. pneumoniae* ATCC700603 [148]	Outcome: 3 log CFU reduction [PS] = 50 µM Light dose: 100 J/cm^2^ (laser *λ* = 630 nm)
22	Toluidine blue (PS**20**) Charge: + 1 *MW* = 270 Da	*E. faecalis* MTCC 2729 and *K. pneumoniae* ATCC700603 [148]	Outcome: 3 log CFU reduction [PS] = 50 µM Light dose: 100 J/cm^2^ (laser *λ* = 630 nm)
23	Azure A (PS**21**) Charge: + 1 *MW* = 256 Da	*E. faecalis* MTCC 2729 and *K. pneumoniae* ATCC700603 [148]	Outcome: 3 log CFU reduction [PS] = 50 µM Light dose: 100 J/cm^2^ (laser *λ* = 630 nm)

**Table 3:** Chemical properties and biological activity of photosensitizers used in vitro assays for inactivation of bacterial biofilms of Gram-negative bacteria

#	Structure	Biofilm strain	Results
1	5-ALA: protoporphyrin IX precursor (PS**9a**) Charge: 0 *MW* = 131 Da	*E. coli* (clinical isolate) and *P. aeruginosa* ATCC 27853 [138]	Outcome: 0 and 3 log CFU reduction, respectively [PS] = 40 mM ALA Light dose: 142 J/cm^2^ (Tungsten Halogen lamps 500 W)
2		*K. pneumoniae* ATCC 700603 [149]	Outcome: 3.1 log CFU reduction [PS] = 10 mM ALA Light dose: 360 J/cm^2^ (150 W xenon lamp *λ* = 400–780 nm)
3	MAL: protoporphyrin IX precursor (PS**9b**) Charge: 0 *MW* = 145 Da	*K. pneumoniae* ATCC 700603 [149]	Outcome: 4.3 log CFU reduction [PS] = 10 mM MAL Light dose: 360 J/cm^2^ (150 W xenon lamp *λ* = 400–780 nm)
4	Porphyrin Mixture FORM (PS**3**) Charge: + 1 to + 4 *MW* = 679 to 900 Da	Bioluminescent *E. coli* [131]	Outcome: ~ 7 log CFU reduction [PS] = 1.0 µM (in combination with 100 mM KI) Light dose: 9 J/cm^2^ (white fluorescent lamp *λ* = 380–700 nm)
5	PS**4** (in polymeric micelles of Pluronic F-127) Charge: + 4 *MW* = 1384 Da	*E. coli* C600 [132]	Outcome: 0.5 log CFU reduction [PS] = 10 µM Light dose: 128 J/cm^2^ (500 W halogen lamp with filter *λ* = 420–1000 nm)
6	TAPP (PS**5**) Charge: 0 to +4 (pH dependent) *MW* = 1019 Da	*P. aeruginosa* (clinical isolate) [133]	Outcome: 3 log CFU reduction [PS] = 30 µM Light dose: 180 J/cm^2^ (Tungsten Halogen lamps 500 W)
7	MgPc-octamorph (PS**10**) Charge: + 8 *MW* = 1690 Da	*P. aeruginosa* K1 [139]	Outcome: 2.4 log CFU reduction [PS] = 1 mM Light dose: 1.9 J/cm^2^ (visible light)
8	ZnPc_iPyr4 (PS**22**) Charge: + 8 *MW* = 1123 Da	Bioluminescent *E. coli* Top10 [150]	Outcome: 2 log CFU reduction [PS] = 40 µM Light dose: 540 J/cm^2^ (Halogen/quartz 250 W lamp)
9	ZnPc_iPyr8 (PS**23**) Charge: + 16 *MW* = 1667 Da	Bioluminescent *E. coli* Top10 [150]	Outcome: 2 log CFU reduction [PS] = 40 µM Light dose: 540 J/cm^2^ (Halogen/quartz 250 W lamp)
10	Curcumin (PS**14**) + Phycocyanin (PS**15**) (in chitosan nanoparticles)	*P. aeruginosa* KY770785.1 [145]	Outcome: 0.9 log CFU reduction [PS] = : 100 µg/ml Light dose: 10 min with 5 W LED lamp
11	Curcumin (PS**14**) (Imobilized in endotracheal tubes)	*E. coli* ATCC 25922 and *P. aeruginosa* ATCC 14502 [144]	Outcome: 0.7 log CFU reduction [PS] = 0.5% w/w Light dose: 50 J/cm^2^ (LED *λ* = 450 nm)
12	Riboflavin (PS**13**) Charge: 0 *MW* = 376 Da	MDR *E. coli* (clinical isolate) [142]	Outcome: 0.2 log CFU reduction [PS] = 50 µM Light dose: n.a. (LED *λ* = 450 nm; 40 W/cm^2^)
13	Rose bengal (conjugate with carbon nanotubules) (PS**24**) Charge: 0/− 1 *MW* = n.a	*E. coli* MCC 2412 [151]	Outcome: 0.4 log CFU reduction [PS] = 50 µg/ml Light dose: 1674 J/cm^2^ (*λ* = 532 nm)
14	SAPYR (PS**16**) Charge: + 1 *MW* = 272 Da	*E. coli* ATCC 25922 [146]	Outcome: 2.9 log CFU reduction [PS] = 500 µM Light dose: 30 J/cm^2^ (gas-discharge lamp *λ* = 380–600 nm)
15	SAPYR-PN-05 (PS**17**) Charge: + 1 *MW* = 407 Da	*E. coli* ATCC 25922 [146]	Outcome: 3.5 log CFU reduction [PS] = 500 µM Light dose: 30 J/cm^2^ (gas-discharge lamp *λ* = 380–600 nm)
16	Methylene blue (PS**18**) Charge: + 1 *MW* = 284 Da	*E. coli* ATCC 25922 [152]	Outcome: 3 log CFU reduction [PS] = 31 µM Light dose: 18 J/cm^2^ (LEDs 625 ± 25 nm)
17		*P. aeruginosa* ATCC 27853 [147]	Outcome: 6 log CFU reduction [PS] = 2.5 mM Light dose: 18 J/cm^2^ (LEDs *λ* = 625 ± 10 nm)
18	Methylene blue (PS**18**) (in dextran capped gold nanoparticles) Charge: + 1 *MW* = 284 Da	*K. pneumoniae* (clinical isolate) [153]	Outcome: 7 log CFU reduction [PS] = 70 µM Light dose: 40 J/cm^2^ (laser *λ* = 660 nm)
19	MB-PMX (PS**25**) Charge: + 1 *MW* = 1723 Da	*E. coli* ATCC 25922 [154]	Outcome: 7 log CFU reduction [PS] = 50 µM Light dose: 288 J/cm^2^ (LED *λ* = 625 nm)

Photosensitizers PS**4**, PS**11**–**15** do not reach a bactericidal effect in the photoinactivation of Gram-positive bacteria under the reported conditions. Identically, photosensitizers PS**4**, PS**9**–**10**, PS**13**–**16**, PS**22**–**24** did not reach a bactericidal effect for Gram-negative bacteria. This set of photosensitizers includes molecules with *MW* > 1000 Da (PS**4**–**5**, PS**10**–**11**, PS**22**–**23**) and molecules that are not positively charged, although they have low molecular weights (PS**9a**, PS**12**–**15**). Photosensitizers PS**9a** (5-ALA), PS**16** (SAPYR), PS**19** (methylene blue derivative), PS**20** (toluidine blue), PS**21** (azure A) are in this list but should be considered as “borderline” cases because they narrowly reach the bactericidal effect but were employed at [PS] ≥ 50 µM. Such high concentrations may be difficult to achieve without toxicity in clinical situations. MAL was not included in this list although it was employed at millimolar concentrations, because of its important bactericidal effect on G− bacteria. 5-ALA and MAL are essential substrates for the biosynthesis PPIX and are efficiently internalized by cells. MAL seems to have a better performance with G− bacteria under the same conditions [149]. The methyl ester of aminolevulinic acid conceals the carboxylate functionality that could prevail at the biological pH and avoiding the presentation of a negative charge may be a factor that contributes to its better performance. Methylene blue (PS**18**) achieves rather impressive log CFU reductions for both G+ and G− bacteria biofilms, however it requires relatively high concentrations. Methylene blue is used in the treatment of methemoglobinemia by intravenous injection (1–2 mg/kg) and it is likely to remain safe at high topical concentrations.

The photosensitizers that reach bactericidal effects are PS**1**–**3**, PS**6**–**8**, PS**17**–**18** and PS**25**. In view of the properties of the other photosensitizers, it is expected that this list of photosensitizers includes positively charged species with *MW* < 1000 Da. This is verified with the exception of PS**6** (*MW* = 1021 Da) and PS**25** (*MW* = 1723 including the polymyxin B moiety). Chlorin e6 (PS**7**) and Photodithazine (PS**8**) achieved bactericidal effects with G+ bacteria at 0.200–2.5 mM concentrations. It is likely that these negatively-charged photosensitizers would require excessively high concentrations to have an effect on G− bacteria.

PS**1** and PS**2** are cationic imidazolyl porphyrins that gave impressive results against G+ and G− planktonic bacteria and against biofilms of G+ bacteria (> 6 log reduction of *S. aureus* biofilms with 5.2 nM @ 5 J/cm^2^ and 1 µM @ 12 J/cm^2^ for PS**1** and PS**2**, respectively), but were not tested against biofilms of G− bacteria. Confocal microscopy revealed that where PS**1** could successfully permeate biofilms, most of PS**2** remained in the planktonic part [82]. As discussed above in Fig. 3, the dense matrix that composes bacterial biofilms hinders the diffusion of antimicrobials towards their interior. This means that amphiphilic and low molecular weight photosensitizers may partition to the biofilms and diffuse more readily inside them. Moreover, the anionic nature of the components of this matrix must also be taken into account. While it can lead to favorable electrostatic interactions with cationic photosensitizers, it is possible that photosensitizers with too many cationic charges are trapped by Coulombic forces in the periphery of biofilm.

PS**3** is a mixture of porphyrins substituted with a different number of pentafluorophenyl and methylpyridynium groups (FORM) and was tested in the photoinactivation of *S. aureus* and *E. coli* biofilms, in combination with KI. It was found that [PS**3**] = 0.1 μM in the presence of [KI] = 100 mM had bactericidal effect even at the modest light dose of 9 J/cm^2^ (Table 2, entry 3) [131]. However, PS**3** is unable to inactivate biofilms in the absence of KI, even at a 20 µM concentration. Hamblin and co-workers showed that using [KI] = 10 mM, which is in the range of KI concentrations in clinically approved products, it was possible to potentiate PDDI with methylene blue of *S. aureus* and *E. coli*, manifested by an additional 2 log units decrease in bacteria survival fraction [60]. This potentiation was explained by the generation of short-lived reactive iodine species (I^⋅^, I_2_^⋅–^) in the reactions between singlet oxygen or hydroxyl radical with iodide ions. Such reactions are expected for photosensitizers in general and it is evident that the good performance of PS**3** benefits from the potentiation with KI. This potentiation helps to explain the prominence of PS**3** in comparison with other photosensitizers in Tables 2 and 3, which did not benefit from the combination with KI.

PS**5** and PS**6** are a porphyrin and the corresponding chlorin, each linked to the same trialkyl amines. Although uncharged at neutral pH, the p*K*_a_ of the conjugate acid is ~ 9–11 and cationization may occur in biological medium [133, 134]. The chlorin is a better sensitizer than the porphyrin when both are excited with light from 350 to 800 nm, as shown by lower concentration of the chlorin for the same effect as the porphyrin. This is readily explained by the intense absorption red light by the chlorin, which leads to more excited states formed and, consequently, to more ROS generated. PS**5** was less effective photoinactivating biofilms of G− bacteria, but it is possible that PS**6** achieves a bactericidal effect for such biofilms at concentration below 50 µM.

SAPYR (PS**16**) and its derivative PS**17** are monocationic photosensitizers, with PS**16** having a pyridinium group and PS**17** an ammonium group with one long alkylic chain. Both photosensitizers were tested in the photoinactivation of *S. mutans*, with PS**16**, requiring a lower concentration (50 µM) than PS**17** (500 µM) to achieve the same 6 log CFU reduction, under a 30 J/cm^2^ light dose [146]. Their efficacy is reversed in *E. coli*, where, under the same drug and light doses, PS**16** and PS**17** achieve 2.9 and 3.5 log CFU reductions, respectively [146]. The performances of both PS**16** and PS**17** are quite remarkable.

Another class of dyes with good performance in the inactivation of biofilms are phenothiazinium dyes (methylene blue, toluidine blue, azure A, rose bengal) [155]. Methylene blue (PS**18**) remains the most interesting photosensitizer of this class with a 6 log CFU reduction of *S. aureus* at 200 μM and 18 J/cm^2^ [147], and bactericidal activity against *E. faecalis* and *K. pneumoniae* G− bacteria [148]. Recently, a methylene blue-polymyxin B (PS**25**) conjugate was reported [154]. Polymyxin B is a potent antibiotic, selective for the inactivation of G− bacteria, and acts through disruption of bacterial membranes through binding to LPS layer. The conjugate yielded a remarkable effect (7 log reduction of *E. coli* in planktonic form at 10 µM concentration and light dose of 6 J/cm^2^). Moreover, a very good result (7 log CFU reduction) for inactivation of *E. coli* in biofilm form (50 µM and 288 J/cm^2^ light dose) was also reported [154].

The differences in light sources and in spectral overlap with the absorption bands of the photosensitizers, the differences in doses and in photosensitizer incubation times, together with the differences in the panels of biofilms tested, recommend caution in the comparison between studies. For example, PDDI efficacy varies by 3 log CFU between ATCC strains and clinical isolates of methicillin-susceptible *S. aureus* (MSSA) and methicillin-resistant *S. aureus* (MRSA) [156]. A similar study recently performed for *E. coli* highlighted the same issue [152]. Nevertheless, the strong bactericidal effects at low doses of PS**1**–**2** and PS**6** (with the caveat that they were not tested against biofilms of G− bacteria), SAPYR and its derivative (PS**16**–**17**), and of methylene blue and its Polymyxin B derivative (PS**18** and PS**25**) suggests that low molecular weight photosensitizers with intrinsic cationic charges or highly basic groups that are protonated at physiological pH, are most promising for PDDI of bacterial biofilms. This can be rationalized considering the barriers for diffusion through the extracellular polymeric matrixes of biofilms and the fact that the bacteria wall is less porous and less fluidic than eukaryotic cells [88]. These factors certainly contribute to the exclusion of large porphyrin derivatives from the short list of the most effective photosensitizers in PDDI of bacterial biofilms and to the inclusion in that list of dyes much smaller than tetrapyrrolic macrocycles. The molecular structures of the most successful photosensitizers in PDDI of bacterial biofilms are dramatically different from those of the photosensitizers presented in Table 1, which reflect the molecular structures preferred for PDT of solid tumors.

The rational presented above should not be interpreted as a discouragement to use neutral or anionic photosensitizers in photodisinfection. They may play important roles in various applications. For example, curcumin (PS**14**) is neutral and 80 µM were required to reduce by 2.0–3.4 log CFU two *S. aureus* strains [143]. However, a significant part of its photodynamic activity was preserved when it was covalently immobilized on the surface of PVC-based endotracheal tubes (0.5% w/w), and immobilized curcumin achieved a 1.3 log CFU reduction of *S. aureus* biofilms [144]. Such photodisinfecting surfaces may help to reduce ventilator-associated secondary infections by MDR bacteria. An example that reached clinical studies is given by neutral tetraphenylporphyrin (TPP) incorporated into a nanofibre textile and applied topically in chronic leg ulcers of 89 patients. After twice daily applications followed by 60 min illumination with white light for a period of 6 weeks, a 35% decrease in wound size was observed [157]. The authors claimed that singlet oxygen, despite being short-lived, can exert an antimicrobial effect in superficial wounds that are in close contact with a photosensitive material. A critical issue in such applications is to obtain a uniform surface modification without photosensitizer leaching over time, to allow for repeated illumination under the same therapeutic conditions.

Bacterial susceptibility to PDDI can vary among different strains [152, 156], but there is no clear correlation with their antibiotic resistance profiles [39]. While one study on clinically isolated MRSA and MSSA showed a tendency for MRSA strains to be less susceptible to PDDI, another report showed that most of the clinically isolated *E. coli* strains used were more susceptible to PDDI than the wild-type ATCC strains [156]. Tables 2 and 3 show that PDDI provides effective treatments for a very broad spectrum of bacteria. The list of bacteria strains susceptible to PDDI in the planktonic form is even more extensive [158]. The underlying mechanisms that confer resistance to antibiotics (e.g., target modification, upregulation of efflux pumps, increased membrane impermeability or production of inactivating enzymes) do not seem to be closely connected with the mechanisms of PS uptake and ROS-mediated oxidative stress.

### Photodisinfection in vivo and ex vivo

In vitro studies with biofilms provide insights into the performance of photosensitizers in PDDI of bacterial infections, but their transition to the clinic requires further studies with biologically relevant models. Tables 4 and 5 list photosensitizers tested in vivo and ex vivo against G+ and G− bacteria, respectively with the intent to bridge the gap to the clinic. Photosensitizer concentration in these studies is either expressed in concentration units or as a drug dose in terms of mass of photosensitizer per body weight of the animal. The latter case refers to systemic administration. In some cases, where a volume is reported, either a local instillation or dropwise addition to the surface of the infection were performed. In general, in vivo experiments require higher photosensitizer concentrations and higher light doses than in vitro studies with biofilms, which may be due to (1) light scattering/absorption by the host tissue, (2) increased difficulty of the photosensitizer molecules to reach the bacteria, (3) reactions of ROS with other biomacromolecules. The doses employed in topical and systemic administrations cannot be directly compared, and we focus our discussion on the photosensitizers used in topical applications.

**Table 4:** Chemical properties and biological activity of photosensitizers used in vivo/ex vivo pre-clinical studies for treatment of infections by Gram-positive bacteria

#	Structure	Model	Results
1	ZnPc-(Lys)_5_ (PS**26**) Charge: 0 to + 5 (pH dependent) *MW* = 1272 Da	In vivo: Sprague–Dawley rats with wound infected by *S. aureus* Xen29 [159]	Outcome: 0.7 log CFU reduction [PS]: 1 µM Light dose: 15 J/cm^2^ (LED *λ* = 680 nm)
2	Arg–Arg–ArgPc (PS**27**) Charge: 0 to + 6 *MW* = 1924 Da	In vivo*:* adult KM mice infected by *S. aureus* [160]	Outcome: 1.6 log CFU reduction [PS]: 50 µM Light dose: 30 J/cm^2^ (500 W halogen lamp with filter *λ* > 610 nm)
3	RLP068 (PS**28**) Charge: + 4 *MW* = 1257 Da	In vivo: BALB⁄c mice with wound infected by MRSA ATCC 43300 [161]	Outcome: ~ 3 log CFU reduction in 2nd day after treatment [PS]: 2 mM Light dose: 60 J/cm^2^ (Laser *λ* = 698 nm)
4	Sinoporphyrin sodium (PS**29**) Charge: − 4 *MW* = 1231 Da	In vivo: female BALB/c mice (5–6 weeks) with burn wound infection by MDR *S. aureus* ATCC 29213 [162]	Outcome: 1 log CFU reduction in 1st and 2nd days; 3 log in in 3rd day after treatment [PS]: 20 µM Light dose: 50 J/cm^2^ (Laser *λ* = 635 nm)
5	5-ALA: protoporphyrin IX precursor (PS**9a**) Charge: 0 *MW* = 131 Da	In vivo: male C57BL/ksj db/db mice with ulcers infected with *S. aureus* MRSA ATCC 33591 [163]	Outcome: 2 log CFU reduction, 7 days after treatment [PS]: 200 mg/kg (1.5 mmol/kg) Light dose: 50 J/cm^2^ (LED *λ* = 410 nm)
6		In vivo: Sprague–Dawley rats CD osteomyelitis model with *S. aureus Xen29* [164]	Outcome: qualitative inhibition of biofilm formation in bone [PS]: 300 mg/kg (2.3 mmol/kg) Light dose: 75 J/cm^2^ (LEDs *λ* = 640 ± 40 nm)
7	Chlorin e6 (PS**7**) Charge: − 3 *MW* = 597 Da	In vivo*:* male BALB/c mice (6 weeks) with subcutaneous infection by *S. aureus* NCTC8532 [165]	Outcome: complete reduction of infection after 5 days [PS]: 10 mg/kg (16 µmol/kg) Light dose: 100 J/cm^2^ (Laser *λ* = 664 nm)
8	Chlorin e6- polyethylenimine conjugate (PS**30**) Charge: positive *MW* = 10,000–25,000 Da	In vivo*:* female BALB/c mice wound infected with MRSA Xen31 [166]	Outcome: 2.7 log CFU reduction [PS]: 400 µM Light dose: 360 J/cm^2^ (Light *λ* = 660 ± 15 nm)
9	Photogem (PS**31**) Charge: − 2 to − 12 *MW* = n.d.	In vivo: Mongolian Gerbils with otitis caused by *S. pneumonia* ATCC 27336 [167]	Outcome: complete reduction of *S. pneumonia* in 87.5% of infections [PS]: 1 mg/ml (20 μl) Light dose: n.d. total energy: 90 J (Laser *λ* = 632 nm)
10	PTMPP (PS**32**) Charge: + 3 *MW* = 663 Da	In vivo: male BALB/c with third degree burns infected with *S. aureus* 8325–4 [168]	Outcome: 1.7 log CFU reduction (7th day) [PS]: 500 µM Light dose: 210 J/cm^2^ (Light *λ* = 635 ± 15 nm)
11	Indocyanine green (PS**33**) Charge: − 2 *MW* = 775 Da	In vivo: rat abrasion wound model infected with MDR *S. aureus* (clinical isolate) [169]	Outcome: 1 log CFU reduction [PS]: 1.2 mM Light dose: 450 J/cm^2^ (Laser *λ* = 808 nm)
12	Hypericin (in nanoparticle formulation) (PS**12**) Charge: 0 *MW* = 504 Da	In vivo: female Wistar rats with wounds infected by MRSA ATCC 6538 [170]	Outcome: disappearance of infection 10 days after treatment [PS]: 0.124 μM Light dose: 23.5 J/cm^2^ (Halogen lamps 20 W)
13	Y1 (PS**34**) Charge: − 1 *MW* = 432 Da	In vivo: adult male ICR mice with skin infection by MRSA [171]	Outcome: 3 log CFU reduction (7th day) [PS]: 2.5 µM Light dose: 30 J/cm^2^ (laser *λ* = 532 nm)
14	Curcumin (PS**14**) Charge: 0 *MW* = 368 Da	In vivo*:* female Balb/C mice infected in the right ear with *S. aureus* ATCC 43300 [172]	Outcome: 2 log CFU reduction on the draining lymph node 72 h after treatment [PS]: 40 mM Light dose: 54 J/cm^2^ (Light *λ* = 450 ± 20 nm)
15		In vivo*:* male Wistar rats with infection by *S. aureus* ATCC 25923 [173]	Outcome: 2 log CFU reduction after treatment [PS]: 1.5% gel (60 µl) Light dose: 60 J/cm^2^ (light *λ* = 450 ± 30 nm)
16	Methylene blue (PS**18**) Charge: + 1 *MW* = 284 Da	Ex vivo: human skin infected with MRSA ATCC 33592 [174]	Outcome: 5.1 log CFU reduction (immediately); 5.9 log reduction (after 24 h) [PS]: 31 µM Light dose: 96 J/cm^2^ (laser *λ* = 670 nm)
17	SAPYR (PS**16**) Charge: + 1 *MW* = 272 Da	Ex vivo*:* human skin colonized by *S. aureus* MRSA ATCC BAA-44 [175]	Outcome: 4 log CFU reduction [PS]: 100 µM Light dose: 60 J/cm^2^ (light *λ* = 380–480 nm)
18	SACUR-3 (PS**35**) Charge: + 4 *MW* = 517 Da	Ex vivo: porcine skin infected with *S. aureus* ATCC 25923 [176]	Outcome: 2 log CFU reduction [PS]: 100 μM Light dose: 34 J/cm^2^ (LED *λ* = 435 ± 10 nm)

**Table 5:** Chemical properties and biological activity of photosensitizers used in vivo/ex vivo pre-clinical studies for treatment of infections by Gram-negative bacteria

#	Structure	Model	Results
1	FS111-Pd (PS**36**) Charge: + 4 *MW* = 938 Da	In vivo: adult female BALB/c mice with wound infection by *E. coli* [64]	Outcome: 4 log CFU reduction initially. Complete inactivation 4 days after treatment [PS]: 50 µM (50 + 20 + 20 µl) Light dose: 80 J/cm^2^ (light *λ* = 415 nm)
2	Photogem (PS**31**) Charge: − 2 to − 12 *MW* = n.d.	In vivo: Mongolian Gerbils with otitis caused by *H. influenza* ATCC 19418 [167]	Outcome: complete reduction of *H. influenza* in 50% of infections [PS]: 1 mg/ml (20 μl) Light dose: n.d. total energy: 90 J (laser *λ* = 632 nm)
3	5-ALA: protoporphyrin IX precursor (PS**9a**) Charge: 0 *MW* = 131 Da	In vivo: Kunming mice infected with *P. aeruginosa* ATCC 27853 [177]	Outcome: 1 log CFU reduction after treatment [PS]: 1.4 M ALA Light dose: 54 J/cm^2^ (light *λ* = 630)
4	Verteporfin (PS**37**) Charge: − 1 *MW* = 718 Da	In vivo: male BALB/c mice with subcutaneous *Mycobacterium bovis* induced granuloma sites [178]	Outcome: 0.7 log CFU reduction, 72 h after treatment [PS]: 0.5 mg/kg (0.7 µmol/kg) Light dose: 60 J/cm^2^ (laser *λ* = 690 nm)
5	EtNBSe (PS**38**) Charge: + 1 *MW* = 445 Da	In vivo: male BALB/c mice with subcutaneous *M. bovis* induced granuloma sites [179]	Outcome: 2 log CFU reduction [PS]: 5.25 mg/kg (11.8 µmol/kg) Light dose: 60 J/cm^2^ cm^2^ (laser *λ* = 635 nm)
6	Chlorin e6-polyethylenimine conjugate (PS**30**) Charge: positive *MW* = 10,000–25,000 Da	In vivo: female BALB/c mice wound infected with *A. baumannii* ATCC BAA 747 [180]	Outcome: 3 log CFU reduction 30 min after treatment. 1.7 log 1–2 days after [PS]: 800–900 µM (50 µl) Light dose: 240 J/cm^2^ (light *λ* = 660 ± 15 nm)
7	Poly-l-lysine-cp6 (PS**39**) Charge: positive *MW* = n.d.	In vivo: female Swiss albino mice with wound infected with *P. aeruginosa* MTCC 3541 [181]	Outcome: 2 log CFU reduction 24 h after treatment [PS]: 200 µM (25 µl) Light dose: 120 J/cm^2^ (light *λ* = 660 ± 25 nm)
8	Tetra-lysine porphyrin (PS**40**) Charge: 0 to + 8 (pH dependent) *MW* = 1188 Da	In vivo: Sprague–Dawley rats with wound infected by mixed bacteria (*E. coli, S. aureus, P. aeruginosa*) [182]	Outcome: 5 log CFU reduction, 7 days after treatment [PS]: 40 µM Light dose: 100 J/cm^2^ (laser *λ* = 650 nm)
		In vivo: adult female BALB/c mice wound infected with multi-resistant *A. baumannii* (clinical isolate) [183]	Outcome: 4 log CFU reduction, 4 days after treatment [PS]: 40 µM Light dose: 50 J/cm^2^ (Laser *λ* = 650 nm)
9	HB: La + 3 (PS**41**) Charge: positive *MW* = n.d.	In vivo: adult female BALB⁄c Mice with burns infected by *P. aeruginosa* (clinical isolate) [184]	Outcome: 2 log CFU reduction in bacteria recovered from blood [PS]: 10 µM (100 µl) Light dose: 24 J/cm^2^ (LED *λ* = 460 ± 25 nm)
10	SACUR-3 (PS**35**) Charge: + 4 *MW* = 517 Da	Ex vivo: porcine skin infected with *E. coli* ATCC 25922 [176]	Outcome: 3 log CFU reduction [PS]: 50 µM Light dose: 34 J/cm^2^ (LED *λ* = 435 ± 10 nm)
11	MB-PMX (PS**25**) Charge: + 1 *MW* = 1723 Da	Ex vivo: porcine skin infected with *E. coli* ATCC 25922 [154]	Outcome: 7 log CFU reduction [PS]: 50 µM Light dose: 288 J/cm^2^ (LED *λ* = 625 nm)
12	Methylene blue (PS**18**) Charge: + 1 *MW* = 284 Da	Ex vivo: cultured human epithelial surfaces infected with MRSA [174]	Outcome: 5 log CFU reduction [PS]: 300 µM with 0.25% chlorhexidine glucose Light dose: 96 J/cm^2^ (LED *λ* = 670 nm)
13		In vivo: female BALB/c mice infected with cecal slurry [185]	Outcome: improved wound healing [PS]: 100 µM Light dose: 24 J/cm^2^ (LED *λ* = 632 nm)

PS**26**–**28** are phthalocyanines with molecular weight above 1200 Da. The CFU reductions obtained with these phthalocyanines were relatively modest, with the possible exception of PS**28**, although it required 2 mM and 60 J/cm^2^ to yield a 3 log CFU reduction in *S. aureus* wound infections in BALB/c mice [161]. Similar CFU reductions were observed with a protoporphyrin IX dimer (Sinoporphyrin, PS**29**) [162] chlorin e6 (PS**7**) [165], a tri-cationic *meso*-substituted porphyrins (PS**32**) [168], polycationic bioconjugates of chlorins (PS**30**) [180] and PS**39** [181], indocyanine green (PS**33**) [169] and curcumin (PS**14**) [172]. None of these photosensitizers fulfill simultaneously the criteria of being cationic and have a low molecular weight. Interestingly, a cationic derivative of curcumin named SACUR-3 (PS**35**) significantly improved the performance of curcumin, which further emphasizes the relevance of positive charges and size to enhance photosensitizer penetration in the bacterial wall [176]. In this respect, the negatively-charged hematoporphyrin derivative photogem (PS**31**) gave surprisingly good results in the photodisinfection of otitis caused by *S. pneumonia* or by *H. influenza* (complete reduction of infection in 87.5% or 50% of the infections, respectively, with 1 mg/ml and 90 J total energy) [167].

Small molecules such as SAPYR (PS**16**) and methylene blue (PS**18**) confirmed in the treatment of ex vivo human skin infections with MRSA the potential for bactericidal effects shown in biofilms. These monocationic photosensitizers achieved 5.1 and 4 log CFU reductions at 31 or 100 μM concentrations and 96 or 60 J/cm^2^, respectively [171, 172, 174–176]. The Polymyxin B derivative of methylene blue (PS**25**) was also used in ex vivo porcine skin infected with *E. coli* and offered a remarkable 7 log CFU reduction [154]. Hypericin (PS**12**) in nanoparticle formulation also proved to be efficient in the reduction of wound infections with MRSA in female Wistar rats at a remarkably low concentration (0.124 μM and 23.5 J/cm^2^) [170]. It is interesting to note that the anionic benzylidene cyclopentanone Y1 (PS**34**) showed a stronger photodisinfection activity than analogous cationic derivatives, achieving a 3 log CFU reduction of wound infections with MRSA. It was argued that this was due to its ability to diffuse through the porin channels to the spheroplast/protoplast of MRSA [171]. This derivative absorbs at 512 nm and has a singlet oxygen quantum yield of only 0.029 [171]. This is a very low value for a photosensitizer and other mechanisms may be relevant for this system.

One of the most successful cases of reducing G− bacterial infections on mice models is that of the non-symmetric tetra-cationic porphyrin complex with Pd(II), FS111-Pd (PS**36**), recently published by Hamblin and co-workers [64]. Photodisinfection with FS111-Pd was more effective (complete inactivation 4 days after treatment with 50 μM photosensitizer concentration and 80 J/cm^2^) than with the corresponding free base macrocycle. The amphiphilicity of the compounds was ensured with three methylpyridinium groups and one pyridinium group linked to a C12 alkyl chain, and Pd (II) improved intersystem crossing rates to the triplet state, increasing its quantum yield and, consequently, that of ROS. However, complexation with heavy metals may not be sufficient to ensure bactericidal effect. Hashimoto [184] used a lanthanum complex of hypocrellin B as photosensitizer to treat burned mice infected with *P. aeruginosa* but 10 μM with a light dose of 24 J/cm^2^ gave only a 2 log CFU reduction in bacteria recovered from blood.

Another interesting case of success is the porphyrin-lysine conjugate with just four units, PS**40**. It reduced by 5 log CFU the bacteria in wounds infected by multiple bacterial strains (*E. coli, S. aureus, P. aeruginosa)*, 7 days after treatment. This study in particular compared the effects of different light doses (12.5, 25, 50 and 100 J/cm^2^) on photodisinfection. It was found that 100 J/cm^2^ was the best light dose for photodisinfection, but this dose worsened wound healing when compared to lower light doses [182]. This demonstrates the importance of fine-tuning PDI protocols to achieve a good compromise between photodisinfection and damage to the host. Furthermore, this photosensitizer was successfully used in the treatment of wounds infected by multi-resistant *A. baumannii* (clinical isolate), where a 4 log CFU reduction was obtained at 40 μM and 50 J/cm^2^ [183].

Overall, there are relatively few cases of topical photodisinfection in vivo where the reduction in CFU achieves the bactericidal level and is sustained for several days. The cases of success are even less common for G− bacterial infections. The most promising photosensitizers are the cationic porphyrin derivatives PS**36** and PS**40**, and the small molecules PS**16** and PS**18** (including the Polymyxin B derivative PS**25**). Their success probably results from efficient interactions between positively-charged photosensitizers with the negatively-charged LPS that are present in outer membrane layer of G− bacteria.

### Clinical studies of photodisinfection

PDDI of oral infections has been extensively covered in several recent reviews [186–191], in view of its interest in dentistry. Most of the studies in this field do not present results in the form of bacterial load reduction. For these reasons, clinical studies on oral photodisinfection are not covered here. It is known that acne responds well to PDT with PpIX precursors and various clinical studies have been published, but the mechanisms of action include anti-inflammatory effects and sebaceous gland inhibition or destruction, in addition to antimicrobial effects [192]. Excluding these clinical applications, the clinical trials involving photoinactivation of bacteria presented in Table 6 correspond only to topical treatments of lower limb infections, often associated with diabetes [193], and to the oral administration of 5-ALA to treat gastritis associated with *Helicobacter pylori* infections. Given these exclusions, the list of photosensitizers is very short. It contains only one phthalocyanine, three phenothiazinium dyes, 5-ALA and MAL.

**Table 6:** Chemical properties and biological activity of photosensitizers used in clinical trials for inactivation of bacteria

#	PS structure/generic name	Condition	Results
1	RLP068 (PS**28**) Charge: + 4 *MW* = 1257 Da	Infected diabetic foot ulcers [194]	Outcome: 2 log CFU reduction compared to placebo, 24 h after one treatment [PS]: 3.5 mM Light dose: 60 J/cm^2^ (laser *λ* = 689 ± 5 nm)
2		Infected diabetic ulcers [195]	Outcome: bacterial CFU count close to 0, after 2nd treatment, in 94% of leg ulcers [PS]: n/a Light dose: 60 J/cm^2^ (*λ* = 630 nm) − 2 × treatments
3		Infected diabetic ulcers [196]	Outcome: significant ulcer reduction, with decrease of bacterial load over the 2 weeks of treatment [PS]: n/a Light dose: 60 J/cm^2^ (*λ* = 630 nm) − 4 × to 6 × treatments
4		Infected diabetic ulcers [197]	Outcome: 40% of the patients completely healed; 28% had ulcer area reduced by > 50%; [PS]: n/a Light dose: 60 J/cm^2^ (*λ* = 630 nm) − 4 × to 16 × treatments
5	PPA-904 (PS**42**) Charge: + 1 *MW* = 533 Da	Chronic leg and foot diabetic ulcers [198]	Outcome: 0.7 log reduction compared to placebo, immediately after treatment. No difference between treatment and placebo groups 24 h after treatment [PS]: ~ 490 µM Light dose: 50 J/cm^2^ (CureLight 01™ *λ* = 570–670 nm)
6	Methylene blue (PS**18**) + toluidine blue (PS**20**) Charge: + 1 *MW* = 284/270 Da	Osteomyelitis (diabetic foot) [199]	Outcome: foot amputation was prevented in 17/18 treatment groups periodontal pathogens versus 0/16 in control group [PS]: ~ 36 mM of each Light dose: 6–30 J/cm^2^ (light *λ* = 400–725 nm)
7	Methylene blue (PS**18**) Charge: + 1 *MW* = 284 Da	Infected diabetic foot ulcers [200]	Outcome: statistically significant decrease of wound area compared to control group [PS]: ~ 312 µM Light dose: 6 J/cm^2^ (light *λ* = 660 nm) − 10 × treatments
8		Infected wounds [201]	Outcome: inactivation of MDR bacteria and wound healing in 5/5 patients [PS]: 31 mM (2 ml) Light dose: 120 J/cm^2^ (light *λ* = 635 nm) − multiple treatments
9	MAL: protoporphyrin IX precursor (PS**9b**) Charge: 0 *MW* = 145 Da	Single case of chronic venous ulceration infected by *S. aureus* and *E. faecalis* [202]	Outcome: clinical improvement and no bacteria detected after treatment [PS]: 160 mg/g MAL cream Light dose: 37 J/cm^2^ (light *λ* = 630 nm) − 4 × treatments
10		Chronic leg ulcers [203]	Outcome: all nine patients had complete ulcer healing after 24 weeks [PS]: 275 µM Light dose: 18 J/cm^2^ (light *λ* = 630 nm) − 8 × treatments
11	5-ALA: protoporphyrin IX precursor (PS**9a**) Charge: 0 *MW* = 131 Da	Chronic skin ulcers in lower limbs infected with *P. aeruginosa* [204]	Outcome: 2 log CFU reduction compared to placebo, 24 h after one treatment [PS]: 1.5 mM Light dose: 80 J/cm^2^ (light *λ* = 630 nm)
12		Infection by *Helicobacter pylori* [205]	Outcome: greatly reduced infection in treated zones of gastric antrum [PS]: ALA 20 mg/kg (0.15 µmol/kg) Light dose: 50 J/cm^2^ (laser *λ* = 410 nm or white light from an Olympus GIF 100 endoscope)

The tetra-cationic phthalocyanine RLP068 (PS**28**) was also evaluated in a single-dose PDDI clinical trial comprising infected foot ulcers in 62 patients aged ≥ 18, with diabetes. This PDDI treatment was used as an add-on to systemic antibiotic administration. The results showed a dose-dependent photosensitizing effect, as higher concentrations had a higher effect in reducing the bacterial load measured in the day after treatment (− 1.92 ± 1.21, − 2.94 ± 1.60, and − 3.00 ± 1.82 log-CFU/ml for 0.10, 0.30, and 0.50% PS concentration vs. − 1.00 ± 1.02 log CFU/ml with placebo). These results illustrate the challenge of clinical treatments with PDDI using large macrocycles [194]. In a pilot study involving 36 patients infected with leg ulcers, two treatments with PS**28** with a 72 h interval resulted in a negative bacterial assay (zero CFU count) in 94% of cases. It is worth noting that bacterial biofilms were present in more than 50% of the cases before treatment, but PDDI treatment successfully eliminated all biofilms [195]. Recently, another pilot study involving multiple-dose treatment using PS**28** over the course of two weeks, also for infected leg ulcers, showed a gradual decrease of microbial load [196]. Another case series involving 22 patients with infected leg ulcers showed a good efficacy of multiple (4× to 16×) treatments using PS**28**. Here, 40% of the patients were considered completely healed, while 28% had ulcer area reduced by > 50%. Additionally, amputation was prevented in 95% of the cases. The authors remark that this PDDI treatment healed infected lesions that had already been treated unsuccessfully with all available methods (local and systemic) and thus prevented amputation, which is considered the last resort treatment [197]. Overall, the clinical studies involving RLP068 (PS**28**) show its potential for the treatment of infected leg ulcers, a fact clearly highlighted by two recent reviews [196, 206]. However, most reports do not comprise randomized controlled trials, which are needed to better assess its benefits over conventional antimicrobial treatments.

The phenothiazinium dye PPA-904 (PS**42**), formulated as a cream (Unguentum M^®^:water 1:2) for topical administration, was investigated in PDDI of infected diabetic ulcers by various types of microorganisms including *S. aureus* and *P. aeruginosa*. PS**42** is similar to methylene blue but has long alkyl chains, which confer more amphiphilicity. The PDDI-treated patients showed a reduction in bacterial load immediately post-treatment but no difference between treatment and placebo groups was observed 24 h after the treatment. The authors found that the bacterial CFU log reduction achieved was similar to those observed in animal models, but lower than those achieved in vitro. Although explanation for this phenomenon demands further investigation, it may be related to the heterogeneity of in vivo infections compared to in vitro experiments with bacteria in planktonic form and interaction of the photosensitizer with endogenous biomolecules, which may lead to singlet oxygen quenching and/or reduction of molecular oxygen available in infected tissue [198].

A mixture of methylene blue (PS**18**) and toluidine blue (PS**20**) was tested in the PDDI treatment of diabetic patients with osteomyelitis in one or more toes. Remarkably, seventeen out of eighteen patients were cured and amputation was prevented, while no effect was observed in sixteen patients of the control group, where amputation had to be performed. It should be noted that at least two of the successfully treated patients had resistant strains of *P. aeruginos*a and *K. pneumonia*. The classic antibiotic treatment for these cases requires intravenous antibiotic therapy often combined with surgical intervention. Such treatments usually require long hospitalization periods with development of MDR strains, which together with the low peripheral circulation and renal insufficiency of these patients, often culminate in amputation. In contrast, PDDI did not require hospitalization or show any relevant side-effects [199]. Phenothiazinium dyes, previously noted for their good performance in PDDI of biofilms and in topical photodisinfection in vivo, were also remarkably successful in this clinical study. Other studies using methylene blue showed a great reduction of wound area in PDDI treated patients with infected wounds [200, 201].

In a single case study, one patient with a chronic venous ulceration infected by *S. aureus* and *E. faecalis* was treated with a topical formulation of MAL. After a total of four PDDI treatments, the patient showed a clinical improvement and no bacteria were detected in the wound [202]. In another study comprising nine patients with chronic leg ulcers, a complete ulcer healing was observed after 8 × sessions of treatment with MAL and irradiation with red light (37 J/cm^2^) [203].

Topical application of 5-ALA on lower limb ulcers caused by *P. aeruginosa* using a 1.5 h of drug-to-light interval, in treatments once a week for two weeks, led to a reduction of the mean ulcer size and improved healing, 7 days after treatment completion. A 2 log CFU reduction 24 h post-treatment was observed in 26 patients [204]. 5-ALA PDI was also used for the inactivation of *H. pylori* in the gastric antrum. Oral administration of 4-ALA was followed 45 min later by illumination of a zone of the gastric antrum either using a laser or endoscopic light. Four hours post irradiation, 85% (laser) and 66% (white light) of biopsies of the illuminated area showed no detectable presence of *H. pylori*. However, bacteria regrowth in the irradiated areas occurred within 48 h of photodisinfection, probably due to reinfection from adjacent areas. Interestingly, maximum uptake and kill of *H. pylori* occurred 20–40 min after oral administration of 5-ALA, whereas the maximum uptake by the gastric mucosa takes 3 h. The selection of the drug-to-light interval allows for selectivity of the antimicrobial effect [205].

In summary, the number of photosensitizers investigated in clinical studies of photodisinfection remains very low, although results obtained in diabetic foot ulcers are very promising. It is expected that the diffusion of the photosensitizer into the wound, the dispersion of light by tissues, the lowered amount of oxygen in poorly irrigated tissues, the competitive reactions of ROS with endogenous biomolecules present in the wound, all combine to make clinical treatments more challenging than pre-clinical studies. Nevertheless, the successful photosensitizers identified in PDDI of biofilms and in vivo justify further efforts to perform clinical trials with cationic photosensitizers of low molecular weight.

## Fungi and biofilms

### General biological structure of fungi

**Fig. 4:**
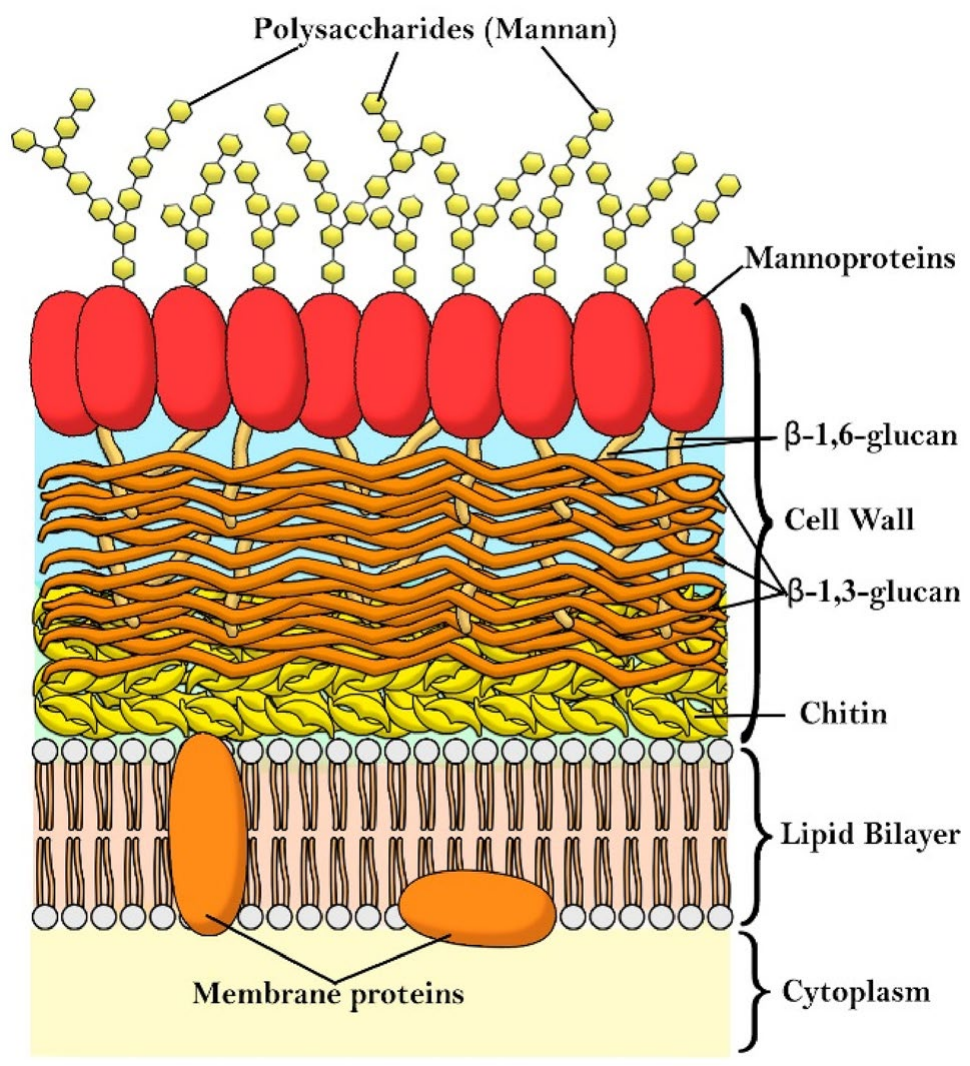
Fungal cell wall (illustrated for *Candida albicans)* [210]

Fungi biological barriers are usually characterized by a lipid bilayer (phosphatidylcholine, phosphatidylethanolamine, phosphatidylserine and ergosterol) [207], enveloped in a cell wall with two components: (1) structural polysaccharide polymers that provide structural rigidity and (2) matrix components that cross-link the polymers and coat the surface, forming an exoskeleton [207]. Overall the cell wall thickness can range from 100 to 400 nm depending on the species [208]. The fungal plasma membrane is unique in the fact that it contains ergosterol, the equivalent of cholesterol in animal cells, constituting one of the primary target of antifungal drugs that are used to treat human mycoses [209]. Relatively to the fungi cell wall, the most prevalent polysaccharide polymers are chitin (polymers of *N*-acetylglucosamine) and glucans (polymers of glucose). In the case of *Candida albicans*, the matrix components consist in mannoproteins, which are essential in protecting the cell against external threats. (Fig. 4) They increase resistance towards antifungal drugs by forming a layer with low permeability and porosity. Additionally, by concealing beneath an immunogenic β-glucan layer, they also reduce the immune response of the host against the fungi [210]. The presence of phosphate groups in mannoproteins side chains confers an overall negative charge to the cell wall [211]. Examples of potential pathogenic fungi include: *Aspergillus fumigatus*, *Cryptococcus neoformans*, *Histoplasma capsulatum* and *Candida albicans*.

### Photoinactivation of fungi

Fungal antimicrobial therapy has already been reviewed by several authors [79, 212–217]. Therefore, here we report only the latest results on biofilms, in vivo pre-clinical and human trials. Table 7 summarizes relevant in vitro biofilm assays published in 2017–2020. Informative reviews specifically focused on earlier work on PDDI of fungi are readily available [218–222].

**Table 7:** Photosensitizer chemical structure/properties and in vitro assays for fungi biofilms inactivation

#	PS structure/generic name	Condition	Results
1	Chlorin e6 (PS**7**) Charge: − 3 *MW* = 597 Da	*C. albicans* ATCC 10231 [223]	Outcome: 0.3 log CFU reduction [PS]: 20 µM Light dose: 40 J/cm^2^ (LED *λ* = 660 nm)
2	Photodithazine (PS**8**) Charge: − 2 *MW* = 581 Da	*C. albicans* (ATCC 90028), *C. glabrata* (ATCC 2001) and *C. tropicalis* (ATCC 4563) [224]	Outcome: 1.0, 1.2 and 1.5 log CFU reduction for *C. glabrata*, *C. albicans* and *C. tropicalis*, respectively [PS]: 154 µM Light dose: 37.5 J/cm^2^ (LED *λ* = 660 nm)
3		*C. albicans* ATCC 90028 [225]	Outcome: 6.1 log CFU reduction after 5× treatment cycles [PS]: 43 µM Light dose: 18 J/cm^2^ (each cycle; LED *λ* = 660 nm)
4	PS**43** Charge: + 1 *MW* = 744 Da	*C. albicans* ATCC 14053, Ca1, Ca2 and Ca3 [226]	Outcome: 6.0, 7.0, 7.0 and 4.0 log CFU reduction, respectively [PS]: 20 µM Light dose: 150 J/cm^2^ (LED *λ* = 410 nm)
5	Porphyrin mixture FORM (PS**3**) Charge: + 1 to + 4 *MW* = 679 to 900 Da	*C. albicans* ATCC 10231 [131]	Outcome: ~ 7 log CFU reduction [PS]: 0.5 µM (in combination with 100 mM KI) Light dose: 9 J/cm^2^ (White fluorescent lamp *λ* = 380–700 nm)
6	Methylene blue (PS**18**) Charge: + 1 *MW* = 284 Da	*C. albicans* ATCC 10231 [227]	Outcome: 0.6 log CFU reduction [PS]: 62.5 µM Light dose: 30 J/cm^2^ (Laser *λ* = 660 nm)
7		*C. auris* (clinical isolate): AR382, AR383, AR385, AR386, AR390 [228]	Outcome: 2.8, 1.5, 7.2, 2.6, 1.6 log CFU reduction, respectively [PS]: 112 µM Light dose: 19.2 J/cm^2^ (LED *λ* = 635 nm)
8	Curcumin (PS**14**) Charge: 0 *MW* = 368 Da	*C. dubliniensis* CBS 7987 [229]	Outcome: 0.5 log CFU reduction [PS]: 40 µM Light dose: 5.3 J/cm^2^ (LED *λ* = 435 nm)
9		*C. albicans* ATCC 90028 [230]	Outcome: 1 log CFU reduction [PS]: 60 µM Light dose: 7.9 J/cm^2^ (LED *λ* = 455 nm)
10	Erythrosin (PS**44**) Charge: − 2 *MW* = 880 Da	*C. albicans* ATCC 18804 [231]	Outcome: 1.1 log reduction of *C. albicans* [PS]: 400 µM Light dose: 42.6 J/cm^2^ (LED *λ* = 532 ± 10 nm)

Anionic photosensitizers such as chlorin e6 (PS**7**) [223] and photodithiazine (PS**8**) [224] gave modest antimicrobial reductions (0.3–1.5 log CFU) at concentrations in the high µM scale with a single PDDI treatment. A significant CFU reduction of *C. albicans* biofilms was only achieved with photodithiazine after 5 successive treatment cycles [225].

PS**43** is a *meso*-di-*trans*-substituted monocationic porphyrin, with a cationic pyridinium group linked to the macrocycle through a C_8_ alkyl chain strengthening its amphiphilic character. This porphyrin showed a remarkable PDDI of *C. albicans* biofilms (4.0–7.0 log CFU reduction) under reasonable drug doses and excitation in the Soret band [226]. This result is in striking contrast with those of the porphyrin mixture FORM (PS**3**), previously mentioned in Tables 2 and 3, which did not achieve any significant CFU reduction of *C. albicans* biofilms without the addition of KI. The presence of KI strongly potentiates PDDI inactivation of fungi, in the same way as it potentiates PDDI of bacteria [131]. It is interesting to remark both the structural similarity of FORM and PS**43** and the widely different results obtained with these photosensitizers. Changing from tetra to di-*trans*-substituted porphyrins seems to improve PDDI of fungi.

Although the example above suggests that PDDI efficacy may increase as the molecular weight of the photosensitizer decreases, the modest photoinactivations observed with methylene blue (PS**18**), curcumin (PS**14**) [229, 230], and erythrosine (PS**44**) [231], advise against generalizations. These small photosensitizers are monocationic, neutral and dianionic, respectively. The dianionic and higher molecular weight photosensitizer of this series was tested at the higher concentrations and did not offer appreciably better results. Methylene blue was tested with five different clinical isolates of *C. auris* and revealed a remarkable strain-dependent effect [228]. Strain dependence and wide variations in performance between structurally-related photosensitizers make it particularly challenging to establish structure–activity relationships. This is clearly an area where more research is urgently needed. Nevertheless, studies with in vivo models, presented in Table 8, help understanding how to optimize PDDI for fungi infections.

**Table 8:** Photosensitizer chemical structure/properties and in vivo pre-clinical assays for fungi inactivation

#	PS structure/generic name	Condition	Results
1	Chloroaluminum phthalocyanine (cationic nano emulsions) (PS**45**) Charge: 0 *MW* = 574 Da	Female swiss mice with oral candidiasis by *C. albicans* ATCC 90028 [232]	Outcome: 2.3 log CFU reduction [PS]: 31.7 µM Light dose: 100 J/cm^2^ (LED *λ* = 660 nm)
2		Female swiss mice with oral candidiasis by *C. albicans* ATCC 90028 [233]	Outcome: 1.4 log CFU reduction after 5 × treatments [PS]: 31.7 µM Light dose: 100 J/cm^2^ (LED *λ* = 660 nm)
3	Photodithazine (PS**8**) Charge: − 2 *MW* = 581 Da	Female Swiss mice with oral infection by *C. albicans* ATCC 96901 and R10/R15 (clinical isolates) [234]	Outcome: 1.96 log CFU reduction for ATCC; 1.15 log CFU reduction for R15; 0 log CFU reduction for R10 [PS]: 100 µM Light dose: 40 J/cm^2^ (LED *λ* = 650–670 nm)
4		Female Swiss mice with oral infection by *C. albicans* ATCC 900283 [235]	Outcome: 3 log CFU reduction 24 h after treatment. Complete remission of all lesions [PS]: 154 µM Light dose: 37.5 J/cm^2^ (LED *λ* = 660 nm)
5	Photogem (PS**31**) Charge: − 2 to − 12 *MW* = n.d.	Mice model with oral candidiasis by *C. albicans* [236]	Outcome: 1.6 log CFU log reduction [PS]: 500 mg/l Light dose: 305 J/cm^2^ (LED *λ* = 455 or 630 nm)
6	TMP-1363 (PS**46**) Charge: + 4 *MW* = 679 Da	Female BALB/c mice with ear infection by *C. albicans* SC5314 [237]	Outcome: 1.7 log CFU reduction [PS]: 220 µM Light dose: 90 J/cm^2^ (Fluorescent lamps *λ* = 575–700 nm)
7	Toluidine Blue (PS**20**) Charge: + 1 *MW* = 270 Da	C57BL/6 mice infected by *T. rubrum* ATCC 28189 [238]	Outcome: 0.7 log CFU reduction [PS]: 7.4 mM gel Light dose: 42 J/cm^2^ (LED *λ* = 630 nm)
8	Methylene blue (PS**18**) Charge: + 1 *MW* = 284 Da	BALB/c female mice tongue (ex vivo) infected by *C. parapsilosis* ATCC 22019 [239]	Outcome: total prevention of biofilm formation in mice tongue [PS]: 1 mM Light dose: 15 J/cm^2^ (LED *λ* = 576–672 nm)
9		Immunosuppressed mice with oral candidiasis by a clinical isolate of *C. albicans* [240]	Outcome: 2.4 log CFU reduction [PS]: 1.5 mM Light dose: 275 J/cm^2^ (laser *λ* = 664 nm)
10		BALB/c mice with oral candidiasis by *C. albicans* CEC 749 [241]	Outcome: almost complete eradication of disease 5 days after treatment [PS]: 10 µl of 1 mM PS solution + 5 µl of 1 M KI solution Light dose: 40 J (LED *λ* = 660 nm)
11	Erythrosin (PS**44**) Charge: − 2 *MW* = 880 Da	Immunosuppressed mice with oral candidiasis by *C. albicans* ATCC 18804 [242]	Outcome: 0.7 log CFU reduction [PS]: 400 µM Light dose: 14.3 J/cm^2^ (LED *λ* = 532 ± 10 nm)
12	Curcumin (PS**14**) Charge: 0 *MW* = 368 Da	Female Swiss mice with oral candidiasis by *C. albicans* ATCC 90028 [243]	Outcome: 1.1 log CFU reduction [PS]: 260 µM Light dose: 37.5 J/cm^2^ (LED *λ* = 455 nm)

Neutral chloroaluminium phthalocyanine (PS**45**) was evaluated as photosensitizer in the treatment of oral candidiasis caused by *C. albicans*. No statistical difference was observed in a DMSO formulation relatively to the non-treated control, but when the phthalocyanine was entrapped in cationic nanoemulsions 2.3 log CFU reduction was observed. In this case, the formulation was crucial in modulating the photosensitizer’s activity, as the cationic nanoemulsions may have reduced photosensitizer aggregation and may have enhanced its interaction with the fungus membrane [232, 233].

Anionic porphyrin photodithiazine (PS**8**) was evaluated in mice with oral infections by four different strains of fluconazole-resistant *C. albicans*. PDDI had no effect in the R10 strain, a noticeable impact on the ATCC 96901 and R15 strains (1.96 and 1.15 log CFU reduction), and a quite remarkable photoinactivation of the ATCC 90028 strain (3 log CFU reduction, 24 h after treatment) [235]. This strengthens the concern that different strains of the same fungi may have different susceptibilities to PDDI, as mentioned for methylene blue in the discussion of Table 7. It cannot be excluded that drug resistance mechanisms (i.e. altered uptake or efflux rates or increase of antioxidant enzymes) have an effect on the final outcome of PDDI [234]. The results obtained with Photogem (PS**32**), which is also an anionic porphyrin photosensitizer, in PDDI of *C. albicans* infecting female Swiss mice tongue (1.6 log CFU) [236] were comparable to those of photodithiazine. The tetracationic porphyrin PS**46** did not perform better than the anionic porphyrin derivatives mentioned above in PDDI of mice ear infected by *C. albicans*. However, confocal microscopy revealed the accumulation of this photosensitizer in the regions of mice ear tissue where fungus cells were located, demonstrating selectivity for the microorganism relatively to the host tissues [237].

Phenothiazinium dyes were also tested for PDDI of fungi infections in mice. Toluidine blue (PS**20**) offered a ~ 0.7 log CFU reduction of *T. rubrum* in a murine model of dermatophytosis, which was slightly higher than the ciclopiroxolamine control [238]. Methylene blue (PS**18**) was tested ex vivo in mice tongue infected with *C. parapsilosis* biofilms and compared with antifungal caspofungin. A 1 mM concentration of methylene blue and 15 J/cm^2^ inhibited biofilm growth even when the treatment was performed 24 h after the beginning of biofilm formation, whereas caspofungin was only efficient when applied before the beginning of biofilm formation [239]. Methylene blue was also used in PDDI oral candidiasis of mice due to an azole-resistant clinical isolated strain of *C. albicans*, and a 2.74 log CFU reduction, corresponding to eradication of *C. albicans*, was achieved at a concentration of ~ 1.5 mM using a light dose of 275 J/cm^2^ [240].

Erythrosin (PS**44**, 400 μM) [242] and curcumin (PS**14**, free or encapsulated in polymeric nanoparticles) [243] were also tested in mice models of oral candidiasis caused by *C. albicans*. The PDDI effect is negligible when curcumin was encapsulated in anionic nanoparticles, and modest otherwise. The best results were obtained with free curcumin (1.1 log CFU reduction at 260 μM and 37.5 J/cm^2^).

The charge of porphyrin derivatives seems to have a less significant effect on PDDI of fungi than of bacteria, especially when the photoinactivation of G− bacteria is considered. Methylene blue remains a major player in this field, although high concentrations must be employed. The clinical trials on fungi photodisinfection, collected in Table 9, provide additional clues on promising photosensitizers. Applications in oral disinfection are not covered in this table because they were recently reviewed by Roomaney et al. [244].

**Table 9:** Chemical properties and biological activity of photosensitizers intended for inactivation of fungi infections in clinical trials

#	PS structure/generic name	Condition	Results
1	Photogem (PS**31**) Charge: − 2 to − 12 *MW* = n.d.	Denture stomatitis (mainly by *C. albicans*, *C. tropicalis* and *C. galabrata*) [245]	Outcome: 1.6 log CFU reduction in palate after 90 days [PS]: 500 mg/l Light dose: 122 J/cm^2^ (LED *λ* = 455 nm) − 6 × sessions
2	5-ALA: protoporphyrin IX precursor (PS**9a**) Charge: 0 *MW* = 131 Da	Single case of chromoblastomycosis by *F. monophora* [246]	Outcome: negative mycological test 4 months after treatment [PS]: 1.5 M ALA Light dose: 96 J/cm^2^ (LED *λ* = 633 ± 10 nm)—4× sessions
3		Interdigital mycosis (*T. mentagrophytes*, *C. albicans*, *T. rubrum*) [247]	Outcome: recovery in 6 out of 9 patients, but recurrence in 4 patients after 4 weeks (1–4 treatments) [PS]: 1.5 M ALA Light dose: 75 J/cm^2^ (filtered white light)
4		Single case of pityriasis versicolor [248]	Outcome: complete healing after 4 weeks (2× treatments) [PS]: 1.5 M ALA Light dose: 70–90 J/cm^2^ (LED *λ* = 630 nm)
5		Two cases of nail onychomycosis [249]	Outcome: complete healing after 6–7× treatments [PS]: 1.5 M ALA Light dose: 100 J/cm^2^ (pulsed laser, *λ* = 630 nm)
6		Nail onychomycosis [250]	Outcome: after 1 year, 43% of the patients were cured [PS]: 1.5 M ALA Light dose: 40 J/cm^2^ (light *λ* = 570–670 nm) − 3× sessions
7	Curcumin (PS**14**) Charge: 0 *MW* = 368 Da	Nail onychomycosis [251]	Outcome: complete healing after 6× sessions [PS]: 41 mM Light dose: 120 J/cm^2^ (LED *λ* = 450 nm)
8	Methylene blue (PS**18**) Charge: + 1 *MW* = 284 Da	Pityriasis versicolor [252]	Outcome: complete cure in 1-month follow-up [PS]: 63 mM Light dose: 37 J/cm^2^ (LED *λ* = 630 nm) 6× in 2 weeks
9		Nail onychomycosis [253]	Outcome: 90% clinical cure rate after treatment; 80% in 12-month follow-up [PS]: 63 mM Light dose: 18 J/cm^2^ (LED *λ* = 630 nm)
10		Nail onychomycosis [254]	Outcome: 100% cure rate for moderate and 64% for severe onychomycosis [PS]: 63 mM Light dose: 36 J/cm^2^ (LED *λ* = 630 nm)
11		Chromoblastomycosis [255]	Outcome: reduction of 80–90% in lesions but no complete healing observed in any of 10 patients [PS]: 700 mM Light dose: 28 J/cm^2^ (LED *λ* = 660 nm) − 6× sessions
12		Leg ulcers infected with *Fusarium oxysporum and P. aeruginosa* [256]	Outcome: complete inactivation of bacteria and fungi and healing after 8 weeks [PS]: 31 mM Light dose: 37 J/cm^2^ (light *λ* = 630 nm)
13		Single case of cutaneous sporotrichosis [257]	Outcome: complete healing after multiple treatments in combination with itraconazole (every 2 weeks for 3 months) [PS]: 31 mM Light dose: 37 J/cm^2^ (LED *λ* = 635 nm)
14	Rose bengal (PS**47**) Charge: 0/− 1 *MW* = 974	Single case of keratitis by MDR *Fusarium keratoplasticum* [258]	Outcome: successful treatment with 2 sessions. No recurrence after 8 month follow-up [PS]: 1 mM Light dose: 2.7 J/cm^2^ (light *λ* = 375 or 518 nm)

The only randomized clinical trial using standard treatment as control where a tetrapyrrolic macrocycle, photogem (PS**31**), was employed as photosensitizer to treat denture stomatitis caused mainly by *C. albicans*, *C. tropicalis* and C. *galabrata*. Photogem-PDDI (six sessions using 500 mg/l of PS concentration and 122 J/cm^2^ light dose) gave a clinical outcome (~ 1.6 log CFU reduction in palate after 90 days follow-up) comparable with standard nystatin antifungal treatment. The authors remarked that PDDI had a better patient compliance than the conventional treatment with nystatin because it requires fewer sessions [245].

Interdigital mycosis was treated with 5-ALA, using a 4 h delay between its topical administration and illumination, and repeating the treatment twice [247]. Only 2 patients had a persistent remission 4 weeks after the last treatment. Similar results were reported in another study involving 10 patients [259]. It is possible that additional treatments would improve the outcome, but ALA-PDT is more expensive and time consuming than other classical treatments.

Pityriasis versicolor is a common chronic superficial infection caused by *Malassezia furfur*. The recommended treatment is oral itraconazole for a long period of time, which is not devoid of systemic side effects. Treatment with topical application of 5-ALA for 4 h followed by irradiation with 70–90 J/cm^2^ allowed the complete clearance of infection 4 weeks after treatment and with no recurrence in the follow-up period [248]. A small clinical study with 5 women treated with topical application of methylene blue for 3 min immediately followed by the light dose, repeated 6 times in a two-week period, led to the clearance of the infection without any recurrence signs 6 months after the start of the treatment.

One of the most explored and successful clinical applications of PDI is in the treatment of nail onychomycosis, as was extensively discussed in several reviews [260–262]. We included in Table 9 some of the most successful examples of PDDI using 5-ALA [249, 250], curcumin [251], and methylene blue [253, 254]. Multiple treatments are necessary. The treatments with 5-ALA were repeated at least weekly and for several weeks; no recurrence of infection was observed after 3- and 6-month follow-ups, but recurrence occurred after 18-months lowering the cure rate. Curcumin required 5–6 PDDI sessions to achieve a complete healing, confirmed by the negative microbiological tests. Methylene blue achieved a 90% cure rate in 12 sessions, which decreased to 80% in a 12-month follow-up due to the recurrence of some infections. This is a better clinical outcome than the group treated with the standard systemic treatment with fluconazole. In another trial with methylene blue, a 100% cure rate was achieved for cases of mild to moderate onychomycosis, while only a 64% cure rate was obtained for severe cases. There is strong clinical evidence that PDDI with small molecules such as 5-ALA, curcumin and methylene blue gives clinical outcomes that are not inferior to conventional anti-fungal treatments of onychomycosis diseases and have favorable patient compliance.

In a study comprising ten patients with chromoblastomycosis treated with methylene blue, although the wound size was reduced, complete healing was not observed for any patient. In most cases, fungi cells were still present in wounds, requiring adjuvant treatment with itraconazole [255]. Methylene blue as PS was also applied to the treatment of leg ulcers infected by *Fusarium oxysporum* and *P. aeruginosa*. A case study showed that PDI completely inactivated both fungi and bacteria, with complete wound healing after 6 months [256]. Methylene blue (one treatment every 2 weeks for 3 months) was also successfully applied in treating a single case of cutaneous sporotrichosis infection, combined with intermittent low doses of the antifungal itraconazole, that did not respond to treatment with MAL [257]. Another single case report refers to the treatment of keratitis by a multi-drug resistant *Fusarium keratoplasticum* using rose bengal [258]. In this particular case, the infection was unresponsive to conventional treatment but a successful clinical outcome was achieved with just two PDDI sessions with no recurrence after 8 months [258]. It is worth noting that this kind of infection may lead to corneal blindness and conventional drug treatments or surgery have a high failure rate.

Clinical translation of PDDI of fungi favors the use of small photosensitizer molecules or the repurposing of photosensitizers employed for other indications. This approach led to conclusive clinical evidence that PDDI is a good clinical option for the treatment of onychomycosis and to encouraging results in other diseases caused by fungi, such as pityriasis versicolor or keratitis. Photosensitizer precursors such as 5-ALA and MAL may not be as cost-effective as pre-formed photosensitizers because they require drug-to-light intervals of 3–4 h.

## Viruses

### Structure of enveloped viruses: example of SARS-CoV-2

**Fig. 5:**
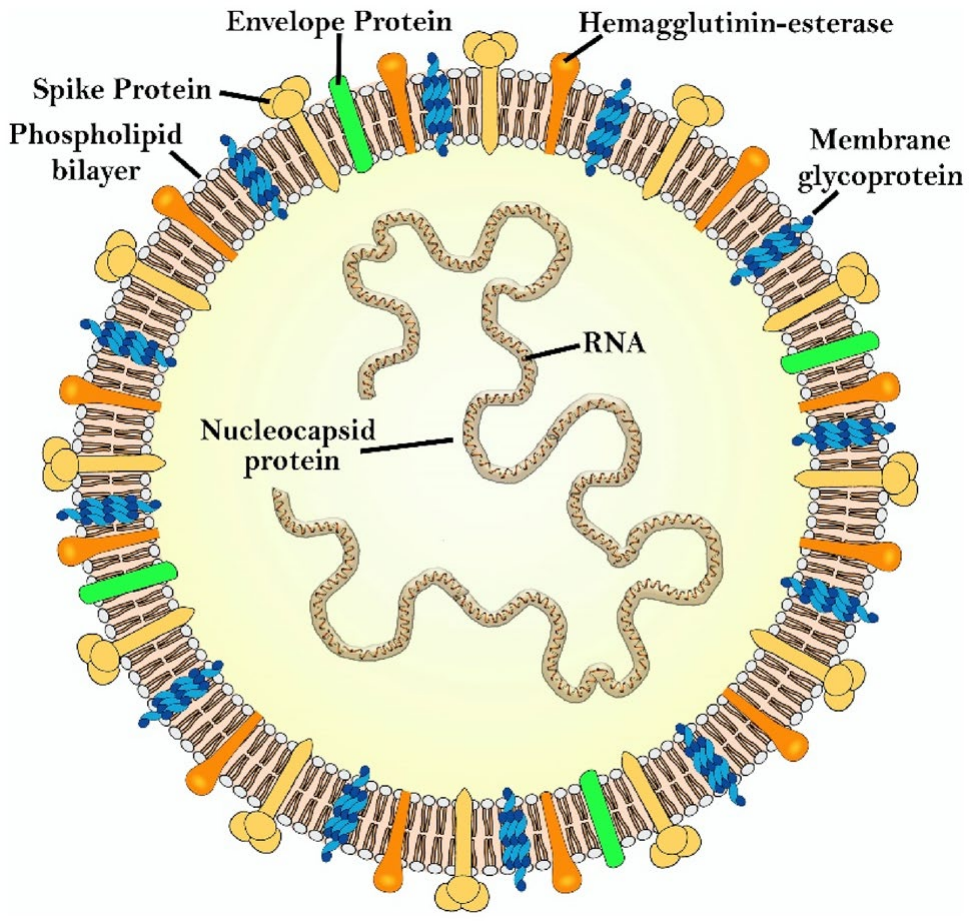
Coronavirus with its lipid envelope containing hemagglutinin-esterase, spike, envelop and membrane proteins, surrounding its positive-sense, single-stranded RNA, embedded in a helical nucleocapsid. Adapted from Graham et al*.* [267].

The representation of a virus envelope in Fig. 5 corresponds to that of coronaviruses, which are a diverse group of single-stranded plus sense RNA virus. These are large enveloped viruses associated with up to 30% of respiratory tract infections in humans. Prior to the COVID-19 pandemic, none of the highly pathogenic zoonotic coronaviruses (SARS-CoV, MERS-CoV, and SARS-CoV-2) or the low-pathogenicity coronaviruses endemic in humans (HCoV-OC43, HCoV-HKU1, HCoV-NL63, and HCoV-229E) had approved therapeutics [263].

It is interesting to point out critical differences between viruses and cells from the point of view of the photodynamic effect. Viruses are the most abundant living entities and can be found in a wide variety of forms. They have in common the ability to infect eukaryotic or prokaryotic cells to force them to produce thousands of copies of the infecting virus. The entry of coronaviruses in cells is mediated by the trimeric transmembrane spike (S) glycoprotein, which comprises a S1 subunit that mediates binding to the host receptor and a S2 subunit that induces fusion of the viral envelope with cellular membranes. S forms an extensive crown decorating the virus surface and is the main target of neutralizing antibodies upon infection [264]. Receptor recognition is the first step of viral infection and is a key determinant of host cell and tissue tropism. The second step is entry of coronavirus into susceptible cells. It requires the concerted action of receptor-binding and proteolytic processing of the S protein to promote virus-cell fusion. Although the process of merging two distinct lipid bilayers into a single one is a thermodynamically favorable reaction, it is associated with a high kinetic barrier. The fusion peptide plays the role of a catalyst in the membrane fusion reaction. It directly interacts with lipid bilayers to disrupt and connect the two opposing membranes [265]. The next step is the delivery of the virus genetic material inside the cell where it is translated to produce viral replication proteins. These proteins selectively bind viral RNA, involve host proteins and lead to recruitment of the viral RNA from translation to replication in viral replication complexes (VCR). New RNAs are released from VCRs, starting a new cycle of translation and replication, become encapsulated and exit the cell [266].

A considerable number of in vitro studies regarding photoinactivation of viruses have been performed, and have already been reviewed by several authors [80, 81, 268–271]. It was found that photoinactivation of viruses possessing a lipid envelope, like the one presented in Fig. 5 as an example, is generally more efficient than of non-enveloped virus. This may imply that the lipid bilayer, or the proteins contained within, are important targets in viral PDI. Indeed, the phospholipids present in the viral membrane can be targeted to prevent viral infections because they are essential to the curvature and fluidity of the membrane. It was shown that, in the presence of light, membrane-binding photosensitizers generate singlet oxygen that oxidizes the C=C double bonds of unsaturated phospholipids leading to *cis*-to-*trans* isomerization and introduction of hydroperoxy (–OOH) groups that result in increased positive curvature and reduced fluidity of the membrane, which affect the ability of viral membranes to undergo fusion [272–274]. The same level of oxidative stress is not toxic to human cells because these benefit from cellular reparative capacities that are absent in static viral membranes. The oxidative stress of PDI may have other targets in addition to unsaturated phospholipids. The most used photodynamic disinfection of blood products for transfusion employs methylene blue, which is known to intercalate with nucleic acids [275].

Virus inactivation with methylene blue is caused by nucleic acid lesions such as strand breaks, cross-linkages or other chemical modifications [276], which interrupts the amplification or reverse transcription of the initial RNA. The inhibition of real-time PCR amplification of treated RNA virus is correlated with the loss of viral infectivity, which is consistent with the damage to RNA being responsible for virus inactivation. Hence, the composition and size of viruses make lipids and nucleic acids as relevant targets for inactivation as proteins.

The COVID-19 pandemic led several authors to propose PDDI as an approach to inactivate SARS-CoV-2 virus and mitigate the effects of the pandemic [53, 277–280]. It was emphasized that PDDI is a good candidate for treating COVID-19 because SARS-CoV-2 is an enveloped RNA virus and these viruses are most sensitive to PDDI. Additionally, the use of light to treat airway related infections is relatively common [281]. In view of the recently published reviews on PDDI of viruses and of the tremendous global impact of the COVID-19 pandemic, below we focus only on PDDI of SARS-CoV-2.

### Photoinactivation of SARS-CoV-2

Just like other SARS-CoV viruses, SARS-CoV-2 is sensitive to ultraviolet light, heat (56 °C for 30 min) and most disinfectants. For example, a 99.99% reduction of the virus on surfaces can be achieved in one minute with exposure to disinfectants such as ethanol (95%), isopropyl alcohol (> 70%), sodium hypochlorite (0.21%), hydrogen peroxide (0.5%) or povidone-iodine (0.23–7.5%) [282]. It is now well established that the mechanism of cellular entry by SARS-CoV-2 is through tight binding to human angiotensin-converting enzyme 2 (hACE2) [263]. The entry receptor hACE2 and the viral entry-associated protease are highly expressed in nasal globlet and ciliated cells, which highlights the potential role of the nasal epithelial cells in initial infection and as possible reservoirs for dissemination within or between individuals [283]. The highest viral loads were found in nasal swabs, rather than throat swabs, in the first few days after the onset of symptoms [284]. Considering that in the early stages of SARS-CoV-2 infection, active virus infection and replication occurs in the apical layer of nasal and olfactory mucosa, nasal lavages with large volumes, e.g., povidone-iodine solution < 5%, have been recommended to limit viral contamination and spreading [285]. Identically, intranasal administration of inhibitors of the fusion between the viral envelope and the host cell were proposed to reduce transmission of SARS-CoV-2 [286]. It was also hypothesized that SARS-CoV-2 binding to the heme groups in hemoglobin is the cause of hypoxia in patients with severe COVID-19, and that the injection of porphyrin-based photosensitizers could block SARS-CoV-2 from binding to hemoglobin, and subsequent illumination could reduce the viral load [287].

Radachlorin and methylene blue are medicinal products in the Russian Federation, and their efficacy to photoinactivate SARS-CoV-2 was investigated to support their repurposing for the treatment of COVID-19. It was found that both radaporfin (0.5–5.0 µg/ml) and methylene blue (1.0–10 µg/ml, i.e., ~ 3–30 µM) protected Vero E6 cell from infection when light doses of 16 and 49 J/cm^2^ were employed [288].

Although COVID-19 was first reported less than two years ago, two clinical studies on PDDI of SARS-CoV-2 have already been published. Riboflavin (PS**13**) was used to test PDDI of SARS-CoV-2 on twenty patients COVID-19 positive, who displayed mild symptoms [289]. After UV/blue light irradiation for 10–20 min of mouth and nose, the viral load decreased significantly in these areas, with 70% of the patients showing a negative PCR test 5 days after treatment, which was accompanied by a reduction of clinical symptoms of the disease. Another trial involved 300 patients with active treatment and 300 with placebo [290]. The active treatment patients had their oral cavity and throat exposed by flushing and gargling to a methylene blue solution (1%) followed by illumination with 72 J/cm^2^ (660 nm), and this procedure was repeated 5 times. The mortality rate was reduced from 3.3% in the placebo group to 0.7% in the active treatment group.

These are promising results but further work is necessary to establish PDDI as a viable therapeutic option for viral infections. It is still not clear if the protection against infection can only be achieved before the virus infects the cells, or if PDDI is also effective when both the photosensitizer molecules and viruses are inside the cells. In the first case a short DLI may favor PDDI of virus without phototoxicity to the cells. However, once the virus infects the cell, it may be very challenging to find selectivity and photoinactivate the virus while sparing the cell.

As mentioned in the Introduction, to achieve maximum efficiency in PDDI, it is always necessary to optimize the combination between the photosensitizer and light source. This aspect is analyzed below.

## Light sources for PDDI

Light is a fundamental part in PDDI and PDT. Despite the crucial role of light in photosensitizer activation, the scientific literature most often describes light sources and light dosimetry separately from the development of photosensitizers and from biological aspects. In this section, we consider light sources in the perspective of photosensitizer activation and address their efficiency, usability and cost effectiveness.

A fundamental, however sometimes misperceived, concept about light activation is that what matters for PDT and PDDI is the number of photons absorbed regardless of their energies [291]. A compound that is suitable for PDDI in general has more than one absorption band in the UV–Vis-NIR window. These absorption bands are connected to the energy levels of the molecule, meaning that light with different energy (or frequency, or wavelength) can excite electrons to different energy levels of the molecule. However, the precursor of singlet oxygen and other ROS is almost invariably the lowest-energy triplet excited state (T_1_) of the photosensitizer [27, 97]. Thus, even if light absorption is occurring to higher singlet energy states (e.g., UV band), the photodynamic reaction will only take place from its T_1_ state, meaning that the same number of photons, whether of UV or visible light, will produce the same number of ROS. This phenomenon is related to Kasha’s rule [292]. For example, light doses in Tables 2, 3, 4, 5, 6, 7, 8 and 9 are expressed in J/cm^2^, but the number of photons at 420 nm or 650 nm is different for the same light dose in these units. A light dose of 10 J/cm^2^ corresponds to 2.0 × 10^19^ photons at 400 nm and 3.3 × 10^19^ photons at 650 nm. It is this number of photons that is related, through the absorption cross section of the photosensitizer and its singlet oxygen quantum yield, to the number of singlet oxygen molecules that will be produced.

**Fig. 6:**
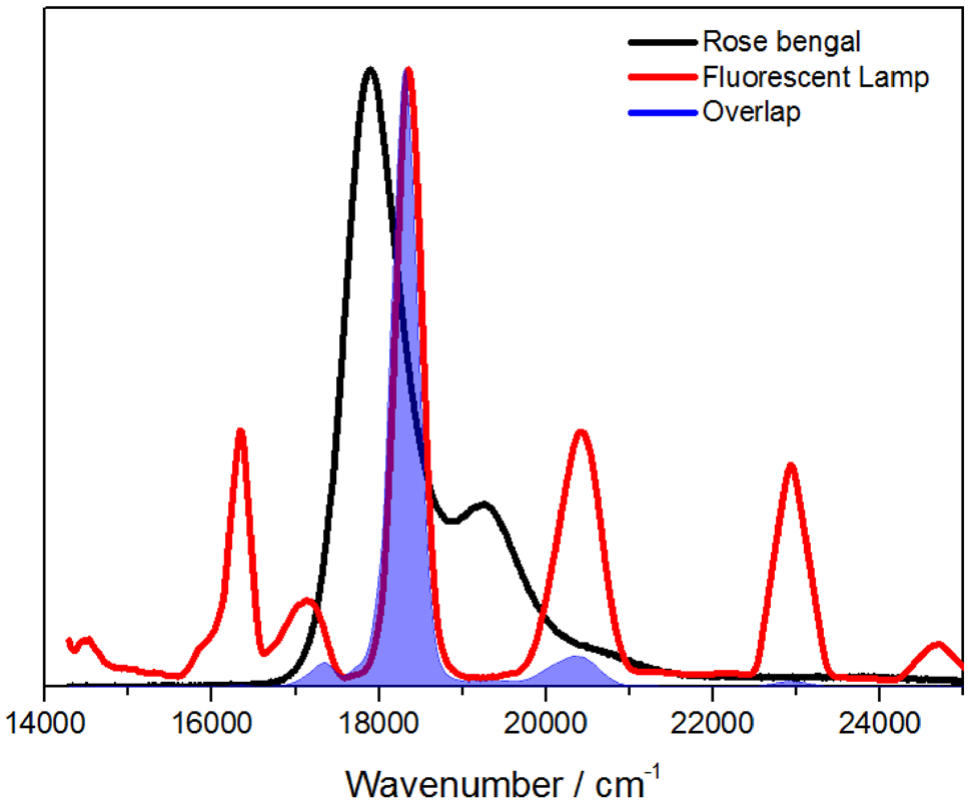
Normalized absorption and emission spectra of Rose Bengal (black) and a fluorescent lamp (red). The overlap resulting in the LDC factor of 0.22 is shown in blue

A proper definition of light dose in PDDI or PDT should consider the number of photons absorbed. This can be achieved by overlapping the absorption spectrum of the photosensitizer with the emission spectrum of the light source. Figure 6 shows the absorption spectrum of Rose Bengal and the emission spectrum of a standard fluorescence lamp used to activate this photosensitizer embedded in an antimicrobial coating [293]. Knowing the total fluence rate in mW/cm^2^ emitted by the lamp, it is possible to calculate the correction factor for the number of photons emitted by the lamp actually absorbed by the photosensitizer, by comparison with the photons absorbed when an ideal monochromatic light source emits at the lowest-energy peak of the photosensitizer absorption spectrum [291]. The overlap of both spectra depicted in Fig. 6 gives the light dose correction (LDC = 0.22) factor that reflects the actual number of absorbed photons [291]. This means that the fluence rate of the light emitted by this fluorescent lamp should be multiplied by 0.22 to give the light dose of an equivalent monochromatic light source. This correction minimizes discrepancies between light doses delivered by different (e.g., broadband, LEDs, laser) light sources.

Light sources in PDDI should be suitable for large field irradiation and anatomically compatible with the target. The wearable cap-like device developed for illumination of the scalp in PDT treatment of actinic keratosis is a good example of the adequacy of the light source to the target [294]. Interesting solutions are now available with fabric-like devices composed of several side emitting optical fibers that can be coupled to a laser device and generate high fluence rates with homogeneous light field. Such devices are very flexible and can adapt to different anatomical structures of the body, allowing homogeneous illumination of large areas [295–297]. Another innovative solution based on quantum dot light-emitting devices (QLEDs), originally employed for displays, was reported [298]. These QLEDs have emission wavelength that can be tuned, being flexible and adaptable, enabling wearable devices for targeted photomedicine.

An interesting example of adaptation of light sources to PDDI targets is the treatment of fungal infections of the nail, which were shown above to be a success case of PDDI. Two light sources devices composed of light emitting diode (LED) arrays were conceived to the treatment of onychomycosis of nails, taking in account the particular anatomo-morphological characteristics of this body structure and the adequacy of a light source for this particular treatment [299]. It was also mentioned above that PDDI has proven its relevance in dentistry, and light sources based on LEDs have been developed for PDDI of the root canal prophylaxis [300]. Another remarkable example of adaptation of light sources is the CE marked device developed by Ondine Biomedical for nasal photodisinfection therapy [301].

The interest in relatively large field illuminations for PDDI of skin infections motivated the development of LED devices tailored for methylene blue (635 ± 11.5 nm) and for sodium magnesium chlorophylin (433 ± 10.5 nm) [302]. This later dye is a food additive and is of interest for the photodisinfection of food. These devices respond to the concern that light sources in clinical settings are likely to require certification as medical devices. The availability of light sources that are certified medical devices will facilitate translation of PDDI to clinic in major markets.

At present, the most appealing applications of PDDI correspond to infections where the microorganisms are less than 3 mm from a surface accessible with a non-invasive light-delivering device. This, however, does not exhaust all possible application of PDDI. Although this review focused on the use of PDI to treat microorganism infections, it is important to emphasize that the photodynamic effect can also play an important prophylactic role. This includes nasal cavity photo-disinfection prior to surgery [303–305] or the disinfection of surfaces and medical devices [306–309]. For example, photosensitizers covalently attached to the surface of endotracheal tubes can prevent bacterial biofilms from colonizing such devices and prevent nosocomial infections [144]. An even more general example is the use of PDDI in environmental disinfection using a antimicrobial coating of Rose Bengal, which actually corresponds to the system illustrated in Fig. 6 [293]. There are vast opportunities to develop combinations of devices with photosensitizers in medical, veterinary, agricultural, food safety and environmental fields.

Finally, it is important to realize that PDDI translation to the clinic will have to prove both that it can have a biocidal effect in a relevant infections disease and that it can be cost effective. The cost of the light source is still regarded as a barrier for market acceptance. However, it must be realized that today any laboratory can build a LED device for a preclinical study with its preferred photosensitizer for less than one thousand euros. LEDs offer relatively narrow emission bands at very affordable prices. They still have the limitation that the fluence rate is in the tens of mW/cm^2^. However, with a powerful photosensitizer with a bactericidal effect at a light dose of 5 J/cm^2^, less than 2 min of illumination at 40 mW/cm^2^ are needed to deliver this light dose.

## Conclusion

PDT of solid tumors has been witnessing the completion of clinical trials and regulatory approval of new photosensitizers in recent years. Chlorin, bacteriochlorin and phthalocyanine derivatives are enjoying the preference of the researchers and clinicians thanks to their intense light absorption in the red and near-infrared. These photosensitizers tend to be relatively large molecules, neutral or anionic, targeting the vasculature or the cell membrane. Using longer drug-to-light intervals, they may also localize inside the cells, with preferential locations in the endoplasmic reticulum, mitochondria or Golgi apparatus. All these photosensitizers are intended for intravenous administration.

PDDI of microorganisms was overlooked for many years but the recognition that antibiotic resistant microorganisms are a major threat to public health and that PDDI can inactivate microorganisms with a lower risk of generating resistance led to a renewed interest in the application of photodynamic effect to treat infectious diseases. The search for new photosensitizers for PDDI was informed by the early recognition that, especially for G− bacteria, cationic photosensitizers should be preferred. With the hindsight of two additional decades of research, it became clear that much of what was learnt about PDDI came from studies where the microorganisms were photoinactivated in the planktonic form. However, it is likely that PDDI will mostly address clinical cases where the microorganisms formed biofilms. Bacteria and fungi attach to surfaces, preferably of dead or poorly irrigated tissue, forming biofilms that offer a protective environment from where the bacteria will detach to colonize other part of the body [122]. The ROS generated when the photosensitizer is excited in the presence of oxygen have a very small diffusion radius compared with the size of biofilms. To be effective against biofilms, the photosensitizers must partition from the aqueous environment to the biofilm matrix and diffuse inside this matrix. The screening of photosensitizers is more meaningful when it evaluates the ability of photosensitizers to have a bactericide effect in biofilms. This is very demanding because biofilms are more difficult to destroy than planktonic bacteria.

An example of our research illustrates the difference between PDDI of bacteria in planktonic and biofilm form. IP-4-Zn (PS**2**) and IP-2-Zn (PS**1**) achieve a 7 log CFU reduction of *S. aureus* with concentrations of 0.1 and 1 µM, respectively, for the same light dose of 2 J/cm^2^ [82]. However, when *S. aureus* forms biofilms, IP-4-Zn gives a 6 log CFU reduction at 1 µM while IP-2-Zn achieves a 7 log CFU reduction at only 0.005 µM (Table 2). This dramatic change in efficacy was shown to result from the rapid diffusion of IP-2-Zn in the biofilm [82]. An excess of positive charges will limit both the partition to the biofilm and the diffusion. The smaller size of IP-2-Zn also favors diffusion.

Relatively few photosensitizers have been used in PDDI of biofilms and an even smaller number reached clinical trials. The photosensitizers in clinical use are mostly repositioning of molecules clinically approved for other indications. Nevertheless, it seems that low molecular weight photosensitizers with intrinsic cationic charges or highly basic groups that are protonated at physiological pH, are most promising for PDDI of biofilms. More research is needed to establish clear structure–activity relations in PDDI of biofilms. This will contribute to a more successful translation of photosensitizers to the clinic. Such research efforts should take into consideration that photosensitizers with negative *n*-octanol:water partition coefficients are likely to stay in the aqueous media rather than partition to the biofilms.

It is interesting to realize that a large fraction of the photosensitizers with better performance in PDDI are not macrocycles. Phthalocyanines and bacteriochlorins are very valuable in PDT of solid tumors because they have molar absorption coefficients *ε* > 100,000 M^–1^ cm^–1^ above 680 nm. However, the depth of treatment of superficial infections will rarely exceed 3 mm and such intense absorptions in the red/near-infrared may not be absolutely required. This is not to say that such macrocycles are not necessary to contribute to the advancement of PDDI. It is rather the recognition that smaller molecules with intense absorptions above 600 nm are major players in PDDI.

The translation of PDDI photosensitizers to clinical settings must be accompanied by the development of light sources. Dedicated light sources will be necessary both to match the lowest-energy absorption band of the photosensitizer and to fit non-invasively the anatomical region of interest. The regulatory pathway for a drug-device combination, or to a medical device with an ancillary medicinal substance (i.e., in the terminology of the European Medicines Agency, a medicine that is incorporated within a medical device where the main mode of action is due to the device), may look fearsome. However, in many clinically-relevant cases PDDI may be positioned as a therapy that does not change the composition of the body, which simplifies the approval of clinical trials.

The control of multidrug-resistant microorganisms will require a wide range of interventions. PDDI should be able to contribute to this control by displacing the use of antibiotics, antifungals or antivirals from localized infections to generalized infections. This will help to avoid, or at least to delay, the development of resistance to antibiotics and increase their useful lifetime. To fulfill this ambition, PDDI must achieve sustained cures at a competitive price. Convincing clinical evidence is now available for periodontal diseases and onychomycosis. Very encouraging results have been reported for infected diabetic foot ulcers and osteomyelitis, as well as for diseases caused by fungi, such as pityriasis versicolor or keratitis. The intense and promising research recently published in this field ensures that other cases of clinical success will appear in the coming years.

It is a pleasure to acknowledge the influence of Mike Hamblin in the development of our views on the best use of the photodynamic effect to inactivate microorganisms.
